# Residential noise exposure and children's emotional and behavioural difficulties in France: a birth cohort study

**DOI:** 10.1016/S2468-2667(26)00128-3

**Published:** 2026-07-21

**Authors:** Eloi Chazelas, Gwendall Petit, Pierre Aumond, Devin Parker, Marie Aline Charles, Maria Melchior

**Affiliations:** a Department of Social Epidemiology, Mental Health and Addictions, Pierre Louis Institute of Epidemiology and Public Health, INSERM U1136, Sorbonne University, Paris, France; b Unité de Mixte de Recherche en Acoustique Environnementale (UMRAE)–Université Gustave Eiffel, CEREMA, UMRAE, Bouguenais, France; c French Institute for Demographic Studies (Ined), French Institute for Medical Research and Health (INSERM), Elfe Joint Unit, Paris, France; d Université Paris Cité, Université Sorbonne Paris Nord, INSERM, Inrae, UMR 1153 Centre for Research in Epidemiology and Statistics Paris University (CRESS), Obstetric, Perinatal and Pediatric Lifecourse Epidemiology (OPPaLE) Team, Paris, France

## Abstract

**Background:**

Over 20% of Europe's population is chronically exposed to transport noise levels considered detrimental to health, yet evidence on its impact on children's mental health remains limited. We aimed to investigate the effect of different sources of residential noise exposure on children's emotional and behavioural difficulties.

**Methods:**

In this birth cohort study, data from the nationally representative French ELFE cohort were analysed from birth to age 10 years (2011–22). We excluded children with missing data on the study's exposures from age 2 months to 5·5 years and on outcome variables at age 10·5 years, twins, children in foster care, and children with profound hearing impairment. Residential exposure to road traffic, aircraft, and railway noise was assessed at age 2 months, 3·5 years, and 5·5 years using GeoClimate and NoiseModelling open-source software and public geographical, traffic, and noise map data. Children's mental health was assessed using the parent-reported Strengths and Difficulties Questionnaire at age 10·5 years. We applied marginal structural models with inverse probability weighting to estimate the effect of time-varying noise exposure through childhood.

**Findings:**

Of the 7914 children included, 4044 (51·1%) were male and 3870 (48·9%) were female. At age 2 months, 2480 (31·3%) children were exposed to low levels (<55 A-weighted decibels [dB(A)]) of road traffic noise exposure, 2772 (35·0%) were exposed to medium levels (55–<60 dB[A]), and 2662 (33·6%) were exposed to high levels (≥60 dB[A]). Persistent exposure to high levels of road traffic noise (≥60 dB[A] across childhood) was associated with higher levels of internalising symptoms at age 10·5 years (adjusted regression coefficient β=0·38, 95% CI 0·14 to 0·63; p=0·003), particularly emotional symptoms (β=0·20, 0·02 to 0·37; p=0·026) and peer relationship problems (β=0·19, 0·06 to 0·32; p=0·009). No significant effects were detected for high exposure to road traffic noise at age 3·5 years. At age 2 months, 161 (2·0%) children were exposed to railway noise of 50 dB(A) or higher. Exposure to railway noise (at least once to ≥50 dB[A] during childhood) was associated with lower levels of internalising symptoms (β=–0·61, 95% CI –1·10 to –0·12; p=0·008), although this association was no longer significant after correction for multiple testing. Exposure to aircraft noise was low, with 185 (2·3%) children exposed to 50 dB(A) or higher at age 2 months, and no significant associations were observed between exposure to aircraft noise and children's emotional and behavioural difficulties.

**Interpretation:**

Although residual confounding cannot be entirely excluded, our results support the need for policies aimed at reducing residential road traffic noise. They require replication in other large-scale cohorts and further investigation of the underlying mechanisms is needed.

**Funding:**

None.

## Introduction

Substantial increases in the incidence, prevalence, and burden of mental disorders have been observed since the COVID-19 pandemic, particularly among young individuals.^1^ These increases are reflected in higher levels of disability, reduced quality of life, and greater health-care and societal costs.^2^ In parallel, environmental noise has become a public health concern in Europe due to widespread exposure and well documented health impacts, including cardiovascular, metabolic, and mental health outcomes. Over 20% of Europe's population is exposed to long-term levels of transport noise considered detrimental to health, as defined by the standard European Environmental Noise Directive threshold day–evening–night level (L_DEN_) of 55 decibels [dB] or higher. This threshold represents long-term daily average noise level measured over 24 h but with penalties added for the evening and night to reflect increased disturbance.^3^ In this context, environmental noise can constitute a stress factor for children. Exposure to acute noise increases physiological stress responses, leading to higher levels of stress hormones such as cortisol and catecholamines. Chronic exposure might cause extended activation of these responses, potentially impacting overall wellbeing, cognition, and mental health.^4^, ^5^ Children might also be particularly sensitive to noise exposure due to their physical, cognitive, emotional, and social development and might have fewer coping strategies than adults.^6^

**Research in context**

**Evidence before this study**

Environmental noise has become a public health concern in Europe due to its widespread exposure and health impacts, including cardiovascular, metabolic, and mental health outcomes. Road traffic is the predominant source of environmental noise, with data from the 2022 Environmental Noise Directive indicating that about 89 million people in Europe are exposed to levels of road traffic noise of 55 A-weighted decibels or higher. Recent trends suggest only modest changes in exposure levels, with slight decreases in road traffic noise in both urban and rural areas, and reductions in aircraft noise (possibly related to reduced air traffic following the COVID-19 pandemic). However, exposure to railway noise increased in urban areas from 2017 to 2022, indicating that overall population exposure to transport-related noise remains substantial. On Dec 15, 2025, we searched PubMed with the search terms (“mental health” OR “problem behavior” OR “development” OR “hyperactivity” OR “emotional”) AND (“noise”) AND (“children” OR “child” OR childhood” OR “teenager”) in the title or abstract. Our search identified 1017 results. Previous studies reported modest and heterogeneous evidence linking residential road traffic noise to higher overall mental health difficulties and hyperactivity and inattention symptoms in children. There was suggestion of a possible association between exposure to railway noise and children's emotional and conduct problems, whereas evidence relative to exposure to aircraft noise was limited and of lower methodological quality. Across reviews, risk of bias was frequently judged as being moderate to high, mainly due to cross-sectional study designs, limited adjustment for confounding factors, and exposure misclassification. Importantly, evidence from large, nationally representative longitudinal cohorts remained scarce. Few studies evaluated distinct noise sources separately, adequately accounted for other environmental exposures such as air pollution, or applied causal inference approaches to address the possibility of time-varying confounding.

**Added value of this study**

This study provides new evidence from a nationally representative birth cohort that persistent exposure to road traffic noise during childhood is associated with increased levels of internalising symptoms at age 10 years. Using marginal structural models, we identified early life and later childhood as potential vulnerability windows, whereas no effects were observed in mid-childhood. No significant associations were found for aircraft noise, and railway noise exposure was unexpectedly associated with lower levels of internalising symptoms, although this association was not significant after correction for multiple testing.

**Implications of all the available evidence**

Our findings add to existing literature regarding the role of road traffic noise as a relevant environmental risk factor of children's mental health difficulties, whereas evidence for exposure to aircraft and railway noise remains less consistent. Timing of exposure might be as important as cumulative exposure, which has implications in terms of prevention strategies. From a public health perspective, these results support policies aimed at reducing residential road traffic noise. They also highlight the need for longitudinal, nationally representative studies that jointly consider multiple noise sources and children's stages of development. Future research should aim to replicate these findings and clarify the underlying mechanisms.

A 2019 meta-analysis reported modest evidence of an association between residential road traffic noise and increased levels of total mental health difficulties and hyperactivity or inattention scores in children.^7^ Previously, a systematic review suggested that road traffic and railway noise could contribute to emotional and behavioural problems, but most studies included were of moderate quality.^4^ The same systematic review reported no association with aircraft noise, but the studies included were of low quality.^4^ More recent cohort studies have shown inconsistent results, highlighting the need for further longitudinal research.^8^, ^9^, ^10^, ^11^, ^12^ Evidence from large, nationally representative cohorts remains poor, with a particular shortage of studies that evaluate distinct noise sources separately^4^ and consider other environmental factors such as air pollution. It also remains unclear whether specific developmental windows during childhood could represent periods of greater vulnerability.

The aim of this study was to investigate the associations between different sources of residential noise exposure and children's emotional and behavioural difficulties, and to explore potential windows of vulnerability across childhood. We used data from a nationally representative cohort of French children followed up from birth to age 10 years, and considered road traffic, aircraft, and railway noise sources.

## Methods

### Study population

The Etude Longitudinale Française depuis l’Enfance (ELFE) birth cohort was initiated to understand how children's environments affect their development, health, and socialisation, from the intra-uterine period to adolescence.^13^ This nationally representative study recruited 18 289 babies born in 2011 from 320 maternity units throughout France. For our study, we used follow-up questionnaire data from telephone interviews with both parents when the child was aged 2 months and with one of either parent when the child was aged 3·5 years, 5·5 years, and 10·5 years.^13^ We excluded children with missing data on the study's exposures from age 2 months to age 5·5 years and on outcome variables at age 10·5 years from our analysis. We also excluded twins (to avoid non-independence of observations), children in foster care (because no geocodes were available to correctly estimate their exposition), and children with profound hearing impairment (appendix p 3). The children included in our analyses were more socioeconomically advantaged than those who were excluded, with higher levels of parental education and higher household socioeconomic positions (appendix pp 4–7). To correct for this non-representativeness and attrition, survey weights were provided by the ELFE study team specifically for the analytical sample of this study. They were constructed following the same procedure as previously described for the 5·5-year wave weights.^14^

Written informed consent was obtained from each parent or the mother alone before enrolment in the original cohort. The regulatory authorities that oversee ethical data collection in France approved the ELFE cohort (Comité de Protection des Personnes number IDFIX-11–024; Comité National Informatique et Libertés number 910504; and CNIS number 2011X716AU).

### Exposure to road traffic, aircraft, and railway noise

Children's home addresses were collected at age 2 months, 3·5 years, and 5·5 years, and geocoded. Children's exposure to environmental noise in the ELFE cohort was estimated at the residential building with receivers modelled around buildings. Road traffic noise was modelled using GeoClimate and NoiseModelling softwares, based on open geographical data and national traffic statistics, with noise emission and propagation computed using the Common Noise Assessment Methods in Europe (CNOSSOS-EU). Railway and aircraft noise exposures were derived from official European strategic noise maps following the same CNOSSOS-EU framework. A harmonised national workflow ensured consistency of noise estimates across the cohort. For each child, different noise sources were expressed in A-weighted decibels (dB[A]), reflecting the frequency sensitivity of human hearing, using the L_DEN_. Full methodological details are provided in the appendix (pp 8–10). The processing workflow and underlying databases are also documented in an open GitHub repository.

### Outcomes

To assess children's emotional and behavioural difficulties at age 10·5 years, parents completed the Strengths and Difficulties Questionnaire (SDQ),^15^ which has previously been validated in French.^16^ Children's symptoms were studied on four negative subscales (five questions for each subscale): hyperactivity and inattention, conduct problems, emotional symptoms, and peer relationship problems. Scores for each subscale ranged from 0 to 10, with higher scores indicating more problematic behaviours or symptoms. The subscales were combined into two broader scales: externalising difficulties (ie, conduct problems plus hyperactivity or inattention symptoms), and internalising difficulties (ie, emotional symptoms plus peer relationship problems). These scores can be studied as either continuous outcomes or categories, and both methods were tested in this study. Validated score thresholds were applied to define so-called normal, borderline, and abnormal categories for each subscale.^17^

### Covariates

Two conceptual directed acyclic graphs in the appendix (pp 11–13) illustrate the road traffic noise analysis and railway and aircraft noise analyses. Two different models were fitted, and the covariates of each model are described in detail in the panel. Briefly, model 1 included the child's characteristics and confounders, while model 2 was further adjusted on environmental and behavioural covariates, as well as additional covariates that are likely to affect the outcome but not the exposure. The environmental covariates were: residential greenness, assessed by the normalised difference vegetation index and described previously;^18^ residential particulate matter with a diameter of 10 μm or less;^19^ walkability index;^20^ and rural or urban environment (<5000 inhabitants *vs* ≥5000 inhabitants). This two-model approach allowed for a minimally adjusted model controlling for confounding alongside a further adjusted model including additional covariates that might improve precision.^22^

Panel **Description of the covariates used in the weighting and outcome models**

**Model 1, based on main child's characteristics and confounders**

*Child characteristics*

• Sex, baseline
• Premature birth, baseline (<37 weeks *vs* ≥37 weeks of amenorrhoea)
• Weight at birth, baseline (<2500 g *vs* ≥2500 g)
• Exact age at which the outcome was reported (months)

*Parental baseline characteristics:*

• Each parent's education level (secondary school or lower; secondary education at high school level; higher education [Baccalauréat + 2–5 years after high school]; Baccalauréat + ≥5 years after high school)
• Each parent's age at child's birth (deciles)
• Each parent's migratory status (not of French nationality; of French nationality descended from one or two immigrant parents; of French nationality descended from French parents)

Parental time-dependent* characteristics:

• Family income per unit of consumption (deciles)
• Parental employment (both parents working, one parent unemployed, both parents unemployed)
• Residential moving since last timepoint (binary)

**Model 2, further adjusted**

Environmental and behavioural covariates, time-dependent* :

• Residential greenness in a 100 m buffer (deciles, assessed by NDVI as described previously)^18^
• Mean PM_10_ (deciles, as described previously)^19^
• Walkability index (terciles, 500 m walkable buffer as described previously)^20^
• Rural or urban environment (<5000 inhabitants *vs* ≥5000 inhabitants)
• Number of siblings living in the house
• Child's total screen time (evaluated at age 3·5 years and 5·5 years, quartiles)

*Further parental baseline covariates:*

• Mother with psychological difficulties during pregnancy (binary)
• Parental postnatal depression (ascertained using the Edinburgh Postnatal Depression Scale dichotomised at a threshold of 13 and summed up for both parents:^21^ neither parent at risk of depression, one parent at risk, or both parents at risk)

As part of the double-robust estimation, outcome models were additionally adjusted for all baseline covariates. For time-dependent covariates, only values measured at 2 months were included in the outcome models to avoid adjusting for variables potentially affected by subsequent exposure. NDVI=normalised difference vegetation index. PM_10_=particulate matter with a diameter of 10 μm or less.

We applied the multiple imputations by chained equations method by fully conditional specification to handle missing covariates data (30 imputed datasets).^23^ For each imputed dataset, we calculated the corresponding weights and implemented regression models before pooling the coefficients. Exposure and outcome variables were not imputed: analyses were restricted to participants with observed data for these variables. This approach was chosen to avoid introducing additional model dependence and potential bias related to the imputation of key variables, particularly outcomes.

### Statistical analysis

We used marginal structural models to estimate the causal effect of time-varying exposure to residential noise on children's internalising and externalising difficulties. Combined with inverse probability weighting, marginal structural models make it possible to estimate the effect of a time-dependent exposure in the presence of time-dependent covariates that might be both confounders and intermediate variables.^24^ Stabilised inverse probability weights were estimated from predicted probabilities of exposure at each timepoint, conditional on past exposure and covariate history. This procedure is described in detail in the appendix (pp 14–24). The weights create a pseudo-population in which exposure is independent of measured confounders, allowing unbiased estimation of the marginal causal effect under the assumption of no residual confounding.^25^, ^26^ We used a double-robust estimation in which the outcome models were further adjusted on baseline covariates (for time-dependent covariates, only the values at the first timepoint were included). Double-robust estimators are consistent if either the weight model or the outcome model is correctly specified, thereby providing additional protection against model misspecification.^27^

For road traffic noise, exposure and covariates were modelled at three timepoints (at ages 2 months, 3·5 years, and 5·5 years). Exposure was categorised into three levels (low exposure [<55 dB(A)_LDEN_], medium exposure [55–60 dB(A)_LDEN_], and high exposure [≥60 dB(A)_LDEN_]). These thresholds were chosen to reflect both policy-relevant cutoffs and the distribution of exposure in the study population. 55 dB(A) corresponds to the threshold commonly used by the European Environment Agency for noise mapping,^28^ whereas 60 dB(A) or higher represents higher exposure levels within the cohort (ie, the third tertile at age 2 months). The reported contrasts (eg, persistent high exposure *vs* persistent low exposure) should be interpreted as counterfactual comparisons of exposure histories over time, corresponding to the expected difference in outcomes if all children had followed one exposure history versus another. First, we estimated the causal effect of persistent high exposure at all three timepoints versus persistent low exposure on each SDQ outcome. To explore specific vulnerability windows, we also estimated the causal effect of being exposed to noise of 60 dB(A) or higher at each specific timepoint while remaining in the category of less than 55 dB(A) at the other two timepoints.

For railway and aircraft noise, given the low prevalence of exposure to railway and aircraft noise in the cohort (2–2·3%), we selected a binary threshold of ever being exposed to 50 dB(A) or higher at any timepoint to balance statistical power with meaningful exposure contrasts. The marginal structural models therefore estimated associations rather than time-specific causal effects, as exposure was not treated as varying over time. Weights were estimated using only baseline covariates for railway and aircraft noise.

We then fitted linear and logistic regression models weighted by stabilised inverse probability and survey weights (to correct for non-representativeness and attrition). The resulting marginal structural model coefficients represent marginal (population-average) differences in SDQ scores comparing the specified exposure patterns. To account for multiple comparisons, we adjusted the p values from model 2 using the Benjamini–Hochberg false discovery rate (FDR) procedure within each table.^29^ R (version 4.3.3) was used for the analyses.

Effect modification by sex, family income, and residential urbanicity was tested within the marginal structural modelling framework using model 2 covariates for road traffic noise only (insufficient exposure prevalence precluded testing for railway and aircraft noise). When a significant interaction was detected, stratified analyses were performed. For stratification by urbanicity, we considered children who remained in the same strata during the whole follow-up period.

We did several supplementary analyses to assess robustness: (1) logistic regressions using validated SDQ subscale thresholds; (2) complete-case analysis; (3) stepwise covariate adjustment to assess time-invariant confounding; (4) additional adjustment for SDQ scores at age 5·5 years to account for pre-existing child difficulties; and (5) quantitative bias analysis including E-values and a housing-focused bias analysis for unmeasured confounding. The full methodology is reported in the appendix (pp 36–46).

### Role of the funding source

There was no funding source for this study.

## Results

The study included 7914 children (4044 [51·1%] male and 3870 [48·9%] female). Table 1 shows the time-dependent characteristics of the study sample. Characteristics by exposure group (ie, road traffic, railway, and aircraft noise) are provided in the appendix (pp 25–33). Overall, parental educational levels were relatively high: 1901 (24·0%) mothers received higher education and 3839 (48·5%) mothers had studied for 5 years or more after high school (table 1). A similar pattern was observed for fathers. Parental employment was also high: in 5861 (74·1%) families, both parents were working at baseline, and the proportion remained stable during follow-up. On average, 2·4% of data on the study covariates were missing, with a maximum of 10·9% for parental postnatal depression.

**Table 1:** Characteristics of the ELFE cohort study population, per timepoint (France, 2011–22; n=7914)

		**Age 2 months**	**Age 3·5 years**	**Age 5·5 years**	**Age 10·5 years**
Sex					
	Male	4044 (51·1%)	..	..	..
	Female	3870 (48·9%)	..	..	..
Birthweight					
	<2500 g	244 (3·1%)	..	..	..
	≥2500 g	7511 (94·9%)	..	..	..
	Missing	159 (2·0%)	..	..	..
Premature birth					
	No, ≤37 weeks of amenorrhoea	7098 (89·7%)	..	..	..
	Yes, >37 weeks of amenorrhoea	700 (8·8%)	..	..	..
	Missing	116 (1·5%)	..	..	..
Mother's age at child's birth, years		31·6 (4·5)	..	..	..
Father's age at child's birth, years		33·9 (5·7)	..	..	..
Mother's educational level					
	Secondary school or lower	267 (3·4%)	..	..	..
	Secondary education (high school)	1859 (23·5%)	..	..	..
	Higher education (Baccalauréat +2–5 years)	1901 (24·0%)	..	..	..
	≥5 years after high school	3839 (48·5%)	..	..	..
	Missing	48 (0·6%)	..	..	..
Father's educational level					
	Secondary school or lower	408 (5·2%)	..	..	..
	Secondary education (high school)	2461 (31·1%)	..	..	..
	Higher education (Baccalauréat +2–5 years)	1469 (18·6%)	..	..	..
	≥5 years after high school	3061 (38·7%)	..	..	..
	Missing	515 (6·5%)	..	..	..
Mother's migratory status					
	Not of French nationality	640 (8·1%)	..	..	..
	French nationality descended from one or two immigrant parents	717 (9·1%)	..	..	..
	Other French mother	6490 (82·0%)	..	..	..
	Missing	67 (0·8%)	..	..	..
Father's migratory status					
	Not of French nationality	175 (2·2%)	..	..	..
	French nationality descended from one or two immigrant parents	1099 (13·9%)	..	..	..
	French nationality descended from French parents	5987 (75·7%)	..	..	..
	Missing	653 (8·3%)	..	..	..
Mother psychological difficulties during pregnancy					
	No	6870 (86·8%)	..	..	..
	Yes	967 (12·2%)	..	..	..
	Missing	77 (1·0%)	..	..	..
Parental postnatal depression risk (EPDS scale)					
	Neither parent at risk	6359 (80·4%)	..	..	..
	One or both parents at risk	690 (8·7%)	..	..	..
	Missing	865 (10·9%)	..	..	..
Income per consumption unit (€ per month)		1814·3 (867·6)	1892·1 (1087·8)	2049·8 (2180·9)	..
Parental employment					
	One parent unemployed	1736 (21·9%)	1358 (17·2%)	1211 (15·3%)	..
	Both parents unemployed	168 (2·1%)	108 (1·4%)	69 (0·9%)	..
	Both parents working	5861 (74·1%)	6023 (76·1%)	5981 (75·6%)	..
	Missing	149 (1·9%)	425 (5·4%)	653 (8·3%)	..
	Mean number of siblings living in the house	0·8 (0·9)	1·1 (0·8)	1·2 (0·8)	..
Road traffic noise exposure					
	<55 dB(A)	2480 (31·3%)	2428 (30·7%)	2459 (31·1%)	..
	≥55 dB(A)	2772 (35·0%)	2941 (37·2%)	2961 (37·4%)	..
	≥60 dB(A)	2662 (33·6%)	2545 (32·2%)	2494 (31·5%)	..
Railway traffic noise exposure					
	<50 dB(A)	7753 (98·0%)	7760 (98·1%)	7914 (100%)	..
	≥50 dB(A)	161 (2·0%)	154 (1·9%)	..	..
Air traffic noise exposure					
	<50 dB(A)	7729 (97·7%)	7737 (97·8%)	7723 (97·6%)	..
	≥50 dB(A)	185 (2·3%)	177 (2·2%)	191 (2·4%)	..
Mean daily screen time (h/day)		..	1·1 (0·9)	1·3 (1·1)	..
Residential moving since last timepoint					
	No	..	6245 (78·9%)	6226 (78·7%)	..
	Yes	..	1534 (19·4%)	1644 (20·8%)	..
	Missing	..	135 (1·7%)	44 (0·6%)	..
Rural or urban environment					
	<5000 inhabitants	2054 (26·0%)	2134 (27·0%)	2268 (28·7%)	..
	≥5000 inhabitants	5793 (73·2%)	5775 (73·0%)	5611 (70·9%)	..
	Missing	67 (0·8%)	5 (0·1%)	35 (0·4%)	..
NDVI		0·5 (0·2)	0·5 (0·1)	0·5 (0·1)	..
PM_10_		23·2 (4·6)	19·2 (3·5)	20·9 (4·6)	..
SDQ externalising symptoms score		..	..	..	4·9 (3·7)
SDQ internalising symptoms score		..	..	..	4·1 (3·1)
SDQ hyperactivity or inattention subscale score		..	..	..	3·3 (2·6)
SDQ conduct problems subscale score		..	..	..	1·6 (1·6)
SDQ emotional problems subscale score		..	..	..	2·7 (2·2)
SDQ peer problems subscale score		..	..	..	1·4 (1·5)
SDQ hyperactivity or inattention subscale score					
	Normal	..	..	..	6247 (78·9%)
	Limit	..	..	..	559 (7·1%)
	Abnormal	..	..	..	1108 (14·0%)
SDQ conduct problems subscale score					
	Normal	..	..	..	5956 (75·3%)
	Limit	..	..	..	987 (12·5%)
	Abnormal	..	..	..	971 (12·3%)
SDQ emotional problems subscale score					
	Normal	..	..	..	5316 (67·2%)
	Limit	..	..	..	947 (12·0%)
	Abnormal	..	..	..	1651 (20·9%)
SDQ peer problems subscale score					
	Normal	..	..	..	6440 (81·4%)
	Limit	..	..	..	701 (8·9%)
	Abnormal	..	..	..	773 (9·8%)

Data are n (%) or mean (SD). Percentages are calculated on the total analytical sample (n=7914). dB(A)=A-weighted decibels. EPDS=Edinburgh Postnatal Depression Scale. NDVI=normalised difference vegetation index. PM_10_=particulate matter with a diameter of 10 μm or less. SDQ=Strengths and Difficulties Questionnaire.

At 2 months, road traffic noise exposure was evenly distributed across the three categories: 2480 (31·3%) children were exposed to low levels (<55 dB[A]), 2772 (35·0%) were exposed to medium levels (55 to <60 dB[A]), and 2662 (33·6%) were exposed to high levels (≥60 dB[A]). Exposure to railway noise was low: 161 (2·0%) were exposed to 50 dB(A) or over at age 2 months. Exposure to aircraft noise was also low, with 185 (2·3%) children exposed to 50 dB(A) or higher at age 2 months. Similar patterns were observed at the other timepoints (table 1). Mean (SD) SDQ scores and the distribution of SDQ categories (both number and percentage) across noise exposure groups are provided in the appendix (pp 34–35), using the same exposure definitions as those applied in the subsequent marginal structural modelling analyses.

Model 1 and model 2 yielded broadly similar estimates; therefore, unless otherwise specified, we have reported results from the further-adjusted model (model 2). Also, p values adjusted using the Benjamini–Hochberg FDR procedure were generally consistent with nominal p values: instances where conclusions differed are reported.

Marginal structural model estimates comparing the association of persistent high (≥60 dB[A]) versus persistent low (<55 dB[A]) exposure to road traffic noise across childhood with children's emotional and behavioural difficulties at age 10·5 years are reported in table 2. Compared with persistent low exposure, persistent high exposure was associated with significantly higher levels of internalising symptoms (adjusted regression coefficient β=0·38, 95% CI 0·14–0·63; p=0·003). When considering SDQ subscales, persistent high exposure was associated with significantly higher levels of both peer relationship problems (β=0·19, 0·06–0·32; p=0·009) and emotional symptoms (β=0·20, 0·02–0·37; p=0·026). The association with emotional symptoms did not remain significant after FDR correction (p=0·052).

**Table 2:** Marginal structural model estimates of the association between persistent high (≥60 dB[A]) versus persistent low (<55 dB[A]) exposure to road traffic noise across childhood and children's emotional and behavioural difficulties at age 10·5 years (ELFE cohort, 2011–22)

		**Model 1**		**Model 2**		
		β (95% CI)	p value	β (95% CI)	p value	Adjusted p value*
SDQ externalising symptoms		0·14 (−0·14 to 0·42)	0·32	0·16 (−0·13 to 0·45)	0·27	0·32
	SDQ hyperactivity	0·12 (−0·07 to 0·32)	0·22	0·13 (−0·07 to 0·34)	0·20	0·30
	SDQ conduct problems	0·02 (−0·10 to 0·14)	0·75	0·03 (−0·10 to 0·16)	0·68	0·68
SDQ internalising symptoms		0·37 (0·12 to 0·61)	0·005	0·38 (0·14 to 0·63)	0·003	0·018
	SDQ emotional symptoms	0·20 (0·02 to 0·37)	0·025	0·20 (0·02 to 0·37)	0·026	0·052
	SDQ peer relationship problems	0·17 (0·04 to 0·30)	0·013	0·19 (0·06 to 0·32)	0·009	0·027

These are marginal estimates of exposure (≥60 dB[A]) at all three timepoints versus persistent low exposure (<55 dB[A]). The estimates represent counterfactual contrasts between exposure histories in the weighted population and should not be interpreted as simple comparisons between observed exposure groups. β=adjusted regression coefficient. dB(A)=A-weighted decibels. SDQ=Strengths and Difficulties Questionnaire.

* p values from fully adjusted model 2, adjusted using the Benjamini–Hochberg false discovery rate procedure.

Table 3 shows the marginal structural model estimates of the association between high (≥60 dB[A]) versus low (<55 dB[A]) exposure to road traffic noise and children's emotional and behavioural difficulties at each timepoint. Compared with persistent low exposure, high exposure at age 2 months was significantly associated with higher levels of peer relationship problems (β=0·23, 95% CI 0·04–0·43; p=0·021), although this association did not remain significant after FDR correction (p=0·086). No significant effects were detected for high exposure at age 3·5 years. High exposure at age 5·5 years was associated with significantly increased levels of externalising (β=0·51, 0·11–0·91; p=0·013) and internalising (β=0·44, 0·10–0·78; p=0·009) symptoms, although these also did not remain significant after FDR correction (p=0·078 for both). When considering SDQ subscales, a significant association was observed between high exposure and hyperactivity symptoms (β=0·36, 0·06–0·65; p=0·024) and peer relationship problems (β=0·28, 0·09–0·47; p=0·005), although these associations did not remain significant after FDR correction.

**Table 3:** Marginal structural model estimates of the association between high (≥60 dB[A]) versus low (<55 dB[A]) exposure to road traffic noise at different timepoints and children's emotional and behavioural difficulties at age 10·5 years (ELFE cohort, 2011–22)

	**Model 1**		**Model 2**		
	β (95% CI)	p value	β (95% CI)	p value	Adjusted p value*
**Exposure at age 2 months only (≥60 dB[A]) *vs* persistent low exposure (<55 dB[A])**					
SDQ externalising symptoms	0·03 (−0·36 to 0·41)	0·90	0·08 (−0·33 to 0·49)	0·70	0·88
SDQ internalising symptoms	0·20 (−0·14 to 0·54)	0·26	0·26 (−0·11 to 0·63)	0·17	0·38
SDQ hyperactivity	0·05 (−0·22 to 0·33)	0·70	0·10 (−0·19 to 0·38)	0·51	0·88
SDQ conduct problems	−0·03 (−0·20 to 0·15)	0·75	−0·02 (−0·21 to 0·17)	0·87	0·92
SDQ emotional symptoms	0·02 (−0·22 to 0·26)	0·87	0·02 (−0·23 to 0·28)	0·86	0·92
SDQ peer relationship problems	0·18 (0·01 to 0·35)	0·043	0·23 (0·04 to 0·43)	0·021	0·086
**Exposure at age 3·5 years only (≥60 dB[A]) *vs* persistent low exposure (<55 dB[A])**					
SDQ externalising symptoms	0·06 (−0·49 to 0·62)	0·82	0·03 (−0·57 to 0·62)	0·93	0·93
SDQ internalising symptoms	−0·06 (−0·49 to 0·37)	0·80	−0·11 (−0·56 to 0·35)	0·65	0·88
SDQ hyperactivity	0·09 (−0·29 to 0·47)	0·65	0·08 (−0·34 to 0·51)	0·70	0·88
SDQ conduct problems	−0·03 (−0·28 to 0·23)	0·84	−0·06 (−0·32 to 0·21)	0·68	0·88
SDQ emotional symptoms	0·02 (−0·28 to 0·33)	0·88	0·06 (−0·27 to 0·38)	0·73	0·88
SDQ peer relationship problems	−0·08 (−0·30 to 0·13)	0·46	−0·16 (−0·40 to 0·07)	0·17	0·38
**Exposure at age 5·5 years only (≥60 dB[A]) *vs* persistent low exposure (<55 dB[A])**					
SDQ externalising symptoms	0·38 (0·01 to 0·74)	0·044	0·51 (0·11 to 0·91)	0·013	0·078
SDQ internalising symptoms	0·43 (0·11 to 0·74)	0·010	0·44 (0·10 to 0·78)	0·009	0·078
SDQ hyperactivity	0·28 (0·02 to 0·55)	0·040	0·36 (0·06 to 0·65)	0·024	0·086
SDQ conduct problems	0·09 (−0·07 to 0·25)	0·266	0·15 (−0·02 to 0·32)	0·092	0·27
SDQ emotional symptoms	0·18 (−0·05 to 0·41)	0·12	0·16 (−0·08 to 0·39)	0·20	0·40
SDQ peer relationship problems	0·24 (0·08 to 0·41)	0·007	0·28 (0·09 to 0·47)	0·005	0·078

These estimates represent counterfactual contrasts between exposure histories in the weighted population and should not be interpreted as simple comparisons between observed exposure groups. β=adjusted regression coefficient. dB(A)=A-weighted decibels. SDQ=Strengths and Difficulties Questionnaire.

* p values from fully adjusted model 2, adjusted using the Benjamini–Hochberg false discovery rate procedure.

Table 4 shows the marginal structural model estimates of the association between childhood exposure to aircraft and railway noise (≥50 dB[A]) at any timepoint and children's emotional and behavioural difficulties. No significant results were observed regarding exposure to aircraft noise. Regarding childhood exposure to railway noise, compared with persistent exposure to less than 50 dB(A), exposure to 50 dB(A) or higher was significantly associated with lower levels of internalising symptoms (β=–0·61, 95% CI –1·10 to –0·12; p=0·008), particularly for emotional difficulties (β=–0·41, –0·73 to –0·08; p=0·017). However, neither association remained significant after FDR correction.

**Table 4:** Marginal structural model estimates of the association between childhood exposure to aircraft and railway noise (≥50 dB[A]) at any timepoint and children's emotional and behavioural difficulties at age 10·5 years (ELFE cohort, 2011–22)

	**Model 1**		**Model 2**		
	β (95% CI)	p value	β (95% CI)	p value	Adjusted p value*
**Aircraft noise**					
SDQ externalising symptoms	0·15 (−0·38 to 0·69)	0·58	0·08 (−0·43 to 0·59)	0·75	0·75
SDQ internalising symptoms	0·49 (−0·00 to 0·98)	0·052	0·45 (−0·05 to 0·94)	0·084	0·29
SDQ hyperactivity	−0·05 (−0·40 to 0·29)	0·76	−0·08 (−0·42 to 0·26)	0·65	0·71
SDQ conduct problems	0·21 (−0·08 to 0·50)	0·16	0·16 (−0·10 to 0·42)	0·23	0·39
SDQ emotional symptoms	0·24 (−0·07 to 0·55)	0·13	0·22 (−0·10 to 0·54)	0·18	0·36
SDQ peer relationship problems	0·25 (−0·05 to 0·54)	0·10	0·23 (−0·06 to 0·51)	0·12	0·29
**Railway noise**					
SDQ externalising symptoms	−0·43 (−1·07 to 0·22)	0·20	−0·37 (−1·07 to 0·32)	0·29	0·39
SDQ internalising symptoms	−0·64 (−1·12 to −0·17)	0·009	−0·61 (−1·10 to −0·12)	0·008	0·096
SDQ hyperactivity	−0·25 (−0·71 to 0·22)	0·30	−0·22 (−0·73 to 0·29)	0·40	0·48
SDQ conduct problems	−0·18 (−0·44 to 0·08)	0·17	−0·15 (−0·42 to 0·11)	0·26	0·39
SDQ emotional symptoms	−0·40 (−0·72 to −0·08)	0·012	−0·41 (−0·73 to −0·08)	0·017	0·10
SDQ peer relationship problems	−0·24 (−0·49 to 0·00)	0·051	−0·21 (−0·47 to 0·05)	0·12	0·29

These estimates represent counterfactual contrasts between exposure histories in the weighted population and should not be interpreted as simple comparisons between observed exposure groups. β=adjusted regression coefficient. dB(A)=A-weighted decibels. SDQ=Strengths and Difficulties Questionnaire.

* p values from fully adjusted model 2, adjusted using the Benjamini–Hochberg false discovery rate procedure.

No significant interactions were found when investigating sex or family income (all p>0·05). A significant interaction with urbanicity was observed (p=0·042 for externalising symptoms and p=0·017 for internalising symptoms), with associations between persistent high road traffic noise and both externalising (β=0·73, 95% CI 0·09–1·36) and internalising symptoms (β=0·84, 0·31–1·36) observed exclusively in rural areas. No significant association was observed in urban areas (appendix p 36).

The results of our supplementary analyses were broadly consistent with the main findings (appendix pp 37–43). The association between exposure to aircraft noise and internalising symptoms was strengthened and reached significance after additional adjustment for previous SDQ scores at age 5·5 years. E-values for significant associations indicated robustness to moderate unmeasured confounding, and housing-focused quantitative bias analysis showed consistently positive bias-adjusted estimates across all scenarios (appendix pp 44–46).

## Discussion

In this cohort study, we found that children experiencing persistently high exposure to road traffic noise from birth to age 5·5 years had higher levels of internalising symptoms at age 10·5 years, particularly in terms of emotional problems and problems with peer relationships. When considering railway noise, children exposed at least once during follow-up had lower levels of internalising symptoms at 10·5 years, particularly emotional difficulties, although the results did not remain significant after correction for multiple testing. No significant associations were observed when considering aircraft noise.

Two systematic reviews have reported modest but consistent associations between exposure to road traffic noise and hyperactivity and inattention, and potential associations with emotional difficulties and behavioural problems, but evidence for aircraft noise remains sparse.^4^, ^7^ More recent cohort studies have shown contrasted results, with some finding no associations in European cohorts,^8^ and others reporting associations with behavioural problems in Brazil,^9^ Switzerland,^10^ and Denmark.^30^ Consistent findings have also been reported outside Europe, including a cross-sectional study of schoolchildren in China, in which exposure to residential road traffic noise was associated with increased total difficulty scores and emotional problems.^11^ Our results extend this literature by suggesting predominant effects of road traffic noise exposure on internalising rather than externalising difficulties, with exposure at age 5·5 years showing stronger associations with externalising symptoms.

Environmental noise constitutes a widespread and growing public health concern rather than a localised issue. Across Europe, a substantial proportion of the population is chronically exposed to transport-related noise at levels exceeding recommended thresholds, with road traffic as the dominant source. Although some recent reductions have been observed, overall exposure remains high, particularly in urban environments, reflecting structural determinants such as urbanisation, transport systems, and land-use patterns. Our findings reinforce the need to consider environmental noise as a systemic and preventable health risk rather than an unavoidable consequence of modern living. From a policy perspective, several interventions are available, including urban planning strategies, traffic reduction, speed regulation, building insulation, and emerging monitoring tools such as noise radars.^31^ Concrete examples of successful implementation include speed limit reductions in Zurich^32^ and roadway redesign in Berlin,^33^ both of which have resulted in reductions in population noise exposure. However, broader implementation faces significant barriers, including high economic costs, industry resistance, and competing urban development priorities, and the EU is currently considered unlikely to meet its 2030 target of a 30% reduction in populations affected by transport noise.^34^ Strengthening regulatory frameworks and integrating noise mitigation into broader environmental and public health policies might be necessary to effectively reduce population exposure.

Noise could affect children's mental health through several mechanisms, including physiological stimulation, annoyance, sleep disturbance, and impaired attention.^5^ Physiological mechanisms involve repeated stimulation of the endocrine system and autonomic nervous system that might lead to catecholamine and cortisol secretion.^4^ Noise annoyance can result in interference with daily activities, sleep, or rest, and might be accompanied by negative responses and stress-related symptoms. Although children might be less vulnerable to noise annoyance than adults, they nevertheless exhibit measurable annoyance responses to environmental noise, particularly in relation to bedroom exposure.^35^, ^36^ Recent evidence has suggested stronger associations for self-reported outcomes than for parent-reported outcomes.^37^ Children are also vulnerable to the cognitive effects of noise exposure, particularly due to their less developed coping strategies,^6^ but epidemiological data regarding cognitive outcomes are scarce, with only moderate evidence regarding reading comprehension and long-term memory.^38^

When examining potential windows of vulnerability, peer relationship problems were similarly associated with high exposure at age 2 months and age 5·5 years. This pattern suggests a potential early-life sensitive period, and a stronger effect when exposure occurs closer to outcome assessment. Associations with externalising symptoms and hyperactivity subscale were observed for exposure at age 5·5 years, suggesting that later childhood exposure could preferentially manifest through externalising behaviours.

Stratification by urbanicity suggests stronger associations of road traffic noise among children living in rural areas. Estimates in urban settings were of similar direction but did not reach significance (p=0·051 for internalising symptoms), which might reflect limited statistical power or reduced exposure variability. In rural settings, baseline environmental noise levels are generally low, thus road traffic noise might represent a more contrasted and disruptive exposure, especially when living in proximity to major roads. Vehicle speed is also probably higher in rural areas. Although speed was considered in our noise modelling, some residual effects related to traffic dynamics cannot be excluded. Future studies should consider acoustic indicators that capture the dynamics of road traffic, such as noise peaks. Other noise sources, such as neighbourhood and recreational noise, could also be expected in urban areas, adding competing sources that could dilute the contribution of road traffic noise. Chronic exposure to background noise in cities might lead to partial habituation (in terms of conscious responses such as annoyance^39^ and cortical and cardiac arousals).^40^ Although we considered contextual parameters such as walkability, urban settings could provide better access to infrastructures that might buffer the negative impact of noise on mental health.

In our study, exposure to railway noise was associated with lower levels of emotional symptoms (contrary to other studies). Railway noise is intermittent and often more predictable than road traffic noise, which has been hypothesised to reduce annoyance. It has historically been considered less annoying than road and aircraft noise,^41^ although more recent studies have shown inconsistent differences between railway and road traffic noise.^42^ Rather than indicating a protective effect, this association might reflect specific residential characteristics of railway environments, with fewer competing noise sources, better infrastructures, and potentially greater use of double-glazed windows. Although inverse probability weighting balanced measured confounders, exposure to railway noise might still act as a marker of specific residential factors associated with lower emotional outcomes that were not considered in this study. Again, contradicting the literature, exposure to aircraft noise in our study was not associated with SDQ when considered continuous. When considering thresholds of SDQ, however, aircraft noise was associated with limited and atypical peer relationship problems. Also, when additionally adjusting for children's externalising and internalising difficulties at age 5·5 years, the association between exposure to aircraft noise and children's internalising symptoms was strengthened and reached significance. This suggests a potential deleterious effect of aircraft noise, which could have been only partially detected here due to the low prevalence of exposure (2·3%).

Our large, nationally representative study yielded results that are generalisable to children living in France, and possibly relevant beyond European settings given broadly consistent findings reported in non-European cohorts.^9^, ^10^, ^11^ The prospective design and causal inference methodology strengthened the plausibility of an effect of exposure to road traffic noise on children's mental health, using time-varying exposure and confounders. However, causality cannot be established from observational evidence alone. The quality of our study also lies in our evaluation of various noise sources and our consideration of other environmental factors that could be highly correlated to noise and which have rarely been considered in previous studies (eg, air pollution, greenness, and walkability).^4^ Outcomes were modelled both continuously and using predefined thresholds, offering a comprehensive understanding of each variable within the general population while also allowing classification into diagnostic categories.

Limitations of the study include imperfect noise modelling precision, which made use of machine learning algorithms for road traffic prediction, not specific to the year of data collection. Also, the low prevalence of exposure to railway and aircraft noise might have reduced our statistical power, leading to imprecise estimates. Although survey weights were constructed specifically for our analytical sample to correct for non-representativeness and attrition, some residual selection bias cannot be entirely excluded, as included participants were more socioeconomically advantaged than those who were excluded, potentially limiting generalisability to more disadvantaged populations. In addition, residual confounding cannot be entirely ruled out. For instance, building elements, such as double glazing, the floor on which the family lived, and bedrooms facing the street, could not be considered. However, the quantitative bias analysis for housing attributes indicated that our results were robust, with bias-adjusted estimates remaining positive across all scenarios and only moderately attenuated, even under extreme assumptions, without approaching the null. Furthermore, as in other epidemiological studies on child mental health, residual confounding by genetic liability or unmeasured familial factors cannot be ruled out. However, a wide range of family-related factors were considered, including parental education, migratory status, income, employment, parental psychological difficulties during pregnancy, and postnatal depression. Additionally, exposure was assessed prospectively across childhood, while mental health outcomes were assessed at age 10·5 years, limiting the likelihood that early child behavioural characteristics substantially influenced long-term residential noise exposure trajectories. Additional adjustment for externalising and internalising difficulties at age 5·5 years suggested that the observed associations are unlikely to be fully explained by early-life vulnerability or pre-existing child characteristics, although this analysis should be interpreted cautiously given the potential for overadjustment. Finally, our double-robust estimation provided protection against model misspecification, and E-values for the main findings suggested that relatively strong unmeasured confounding would be required to fully explain the observed associations. However, although multiple testing was accounted for, effect sizes remained modest, and confidence intervals were often close to the null, suggesting cautious interpretation, particularly for railway and aircraft noise estimates.

Although our findings need replication in other large-scale cohorts and further investigation of the underlying mechanisms, they support the implementation of policies aimed at reducing environmental noise exposure, particularly from road traffic.

### Contributors

### Data sharing

To access data, researchers can make a request to the ELFE data access committee. Researchers who are interested in accessing ELFE questionnaires or data can refer to https://elfe.site.ined.fr/en/cote-recherche/acces-aux-donnees-et-questionnaires. Codes used to construct the noise indicators are available through open repositories (https://github.com/Universite-Gustave-Eiffel/).

For the **GitHub repository** see https://github.com/Universite-Gustave-Eiffel/OpenDataNoiseMap

## Declaration of interests

We declare no competing interests.

## Footnotes

* Modelled at three timepoints (at ages 2 months, 3·5 years, and 5·5 years). Time-dependent covariates were used for only the road traffic noise model. For railway and aircraft noise models, these covariates were considered at baseline only.
