# Supplementary material for: Residential noise exposure and children's emotional and behavioural difficulties in France: a birth cohort study
Source: Lancet Public Health. 2026 Jul 21;11(8):e543–54. doi: 10.1016/S2468-2667(26)00128-3 (PMC13752250; doi:10.1016/S2468-2667(26)00128-3)
Supplement: Supplementary file 1 [file mmc1.pdf]

THE LANCET
Public Health

Supplementary appendix

This appendix formed part of the original submission and has been peer reviewed. We post it as supplied by the authors.

Supplement to: Chazelas E, Petit G, Aumond P, Parker D, Charles MA, Melchior M. Residential noise exposure and children's emotional and behavioural difficulties in France: a birth cohort study. *Lancet Public Health* 2026; **11:** e543–54.

Section 1: Flowchart 3
Section 2: Comparison between included and excluded participants 4
Supplementary Table 1: Comparison between included and excluded participants (ELFE cohort study, France, 2011-2022) 4
Section 3: Modelling of road traffic, aircraft and railway noise 8
Section 4: Conceptual DAGs 11
Supplementary Figure 1: Conceptual DAG of the road traffic noise analyses. 12
Supplementary Figure 2: Conceptual DAG of the railway and aircraft traffic noise analyses. 13
Section 5: Inverse Probability Weights estimation 14
Supplementary Table 2: Road models ASMD report before and after weighting 15
Supplementary Figure 3: Histogram road model 2 (mean of weights= 1.012): 20
Supplementary Table 3: Aircraft and railway models ASMD reports before and after weighting 21
Supplementary Figure 4: Histogram aircraft Model 2 (mean of weights = 0.994): 24
Supplementary Figure 5: Histogram railway Model 2 (mean of weights = 0.993): 24
Section 6: Characteristics of the study population by exposure group at 2 months 25
Supplementary Table 4: Characteristics of the study population by road traffic noise at 2 months (ELFE cohort study, France, 2011-2022) 25
Supplementary Table 5: Characteristics of the study population by railway noise at 2 months (ELFE cohort study, France, 2011-2022) 28
Supplementary Table 6: Characteristics of the study population by aircraft noise at 2 months (ELFE cohort study, France, 2011-2022) 31
Section 7: Description of SDQ scores across exposure categories. 34
Supplementary Table 7: Mean (SD) SDQ scores across traffic noise exposure groups (ELFE cohort study, France, 2011–2022) 34
Supplementary Table 8: Distribution of SDQ categories according to traffic noise exposure groups at age 10.5 years (ELFE cohort study, France, 2011-2022) 35
Section 8: Stratification by rural/urban area 36
Supplementary Table 9: Marginal structural model estimates of the effect of persistent high (≥60 dB(A)) versus low (<55 dB(A)) road traffic noise exposure across childhood on children's emotional and behavioral difficulties at 10·5 years – stratification by rural/urban area (ELFE cohort, 2011–2022) 36
Section 9: Analyses considering thresholds of SDQ 37

Supplementary Table 10: Marginal structural model estimates of the effect of persistent high (≥60 dB(A)) versus low (<55 dB(A)) road traffic noise exposure across childhood on children's emotional and behavioral difficulties at 10·5 years - considering thresholds of SDQ (ELFE cohort, 2011–2022)....................37

Supplementary Table 11: Marginal structural model estimates of the effect of childhood exposure to aircraft and railway noise (≥50 dB(A)) at any timepoint and children's emotional and behavioral difficulties at 10·5 years - considering thresholds of SDQ (ELFE cohort, 2011-2022)....................38

Section 10: Complete case analyses 39

Supplementary Table 12: Marginal structural model estimates of the effect of persistent high (≥60 dB(A)) versus low (<55 dB(A)) road traffic noise exposure across childhood on children's emotional and behavioral difficulties at 10·5 years – complete case (ELFE cohort, 2011–2022)....................39

Supplementary Table 13: Marginal structural model estimates of the effect of childhood exposure to aircraft and railway noise (≥50 dB(A)) at any timepoint and children's emotional and behavioral difficulties at 10·5 years – complete case (ELFE cohort, 2011-2022)....................40

Section 11: Stepwise regression models....................41

Supplementary Table 14: Associations between exposure to residential noise and children's emotional and behavioral difficulties at 10·5 years – stepwise regression models (ELFE cohort, 2011-2022)....................41

Section 12: Additionally-adjusted models for externalizing and internalizing difficulties at 5.5 years....................42

Supplementary Table 15: Marginal structural model estimates of the effect of persistent high (≥60 dB(A)) versus low (<55 dB(A)) road traffic noise exposure across childhood on children's emotional and behavioral difficulties at 10·5 years - Further-adjusted model 2 (ELFE cohort, 2011–2022)....................42

Supplementary Table 16: Marginal structural model estimates of the effect of childhood exposure to aircraft and railway noise (≥50 dB(A)) at any timepoint and children's emotional and behavioral difficulties at 10·5 years - Further-adjusted model 2 (ELFE cohort, 2011-2022)....................43

Section 13: Quantitative bias analysis for unmeasured confounding. 44

Supplementary Figure 6: E-value for the association between road traffic noise and abnormal peer relationship problems SDQ subscale 44

Supplementary Table 17: E-values based on MSM estimates of childhood exposure to road, aircraft and railway noise and children's emotional and behavioral difficulties at 10·5 years - considering thresholds of SDQ (ELFE cohort, 2011-2022) 45

References....................47

Section 1: Flowchart

18,329 participants included in ELFE cohort in 2011.

↓ Exclusion of 8,585 participants without SDQ data at age 10.5.

9,744 participants with complete SDQ at age 10.5.

↓ Exclusion of 1,717 participants with incomplete exposure variables (from 2 months to 5.5 years).

8,027 participants with complete exposure variables (from 2 months to 5.5 years).

↓ Exclusion of 102 twins, 6 children with profound hearing impairment, 5 children placed in foster care.

7,914 participants included.

Section 2: Comparison between included and excluded participants

Supplementary Table 1: Comparison between included and excluded participants (ELFE cohort study, France, 2011-2022)

Characteristics		Excluded participants (n=10415)	Included participants (n=7914)
Sex	Male	5327 (51.1)	4044 (51.1)
	Female	4989 (47.9)	3870 (48.9)
	Missing	99 (1.0)	0 (0.0)
Birth weight	<2500 g	677 (6.5)	244 (3.1)
	≥2500 g	9396 (90.2)	7511 (94.9)
	Missing	342 (3.3)	159 (2.0)
Premature birth	No, <=37 weeks of amenorrhea	8715 (83.7)	7098 (89.7)
	Yes, >37 weeks of amenorrhea	1424 (13.7)	700 (8.8)
	Missing	276 (2.7)	116 (1.5)
Mean (SD) mother's age at child's birth		29.9 (5.4)	31.6 (4.5)
Mean (SD) father's age at child's birth		32.8 (6.6)	33.9 (5.7)
Mother's educational level at 2 months	Secondary school or lower	1043 (10.0)	267 (3.4)
	Secondary education (high school)	3633 (34.9)	1859 (23.5)
	Higher education (Bac + 2 to +5)	1692 (16.2)	1901 (24.0)
	≥5 years after high school	2185 (21.0)	3839 (48.5)
	Missing	1862 (17.9)	48 (0.6)
Father's educational level at 2 months	Secondary school or lower	742 (7.1)	408 (5.2)
	Secondary education (high school)	3000 (28.8)	2461 (31.1)
	Higher education (Bac + 2 to +5)	1006 (9.7)	1469 (18.6)
	≥5 years after high school	1486 (14.3)	3061 (38.7)
	Missing	4181 (40.1)	515 (6.5)
Mother's migratory status	Not of French nationality	1040 (10.0)	640 (8.1)
	French nationality descended from 1 or 2 immigrant parents	1005 (9.6)	717 (9.1)
	Other French Mother	6515 (62.6)	6490 (82.0)
	Missing	1855 (17.8)	67 (0.8)
Father's migratory status	Not of French nationality	566 (5.4)	175 (2.2)
	French nationality descended from 1 or 2 immigrant parents	1186 (11.4)	1099 (13.9)
	Other French Father	4518 (43.4)	5987 (75.7)

	Missing	4145 (39.8)	653 (8.3)
Mother psychological difficulties during pregnancy	No	8889 (85.3)	6870 (86.8)
	Yes	1297 (12.5)	967 (12.2)
	Missing	229 (2.2)	77 (1.0)
Parental postnatal depression risk (EPDS scale)	Neither parent at risk	4852 (46.6)	6359 (80.4)
	One or both parent at risk	623 (6.0)	690 (8.7)
	Missing	4940 (47.4)	865 (10.9)
Income per consumption unit at 2 months (€/month)		1458.8 (1102)	1814.3 (867.6)
Parental employment at 2 months	One parent unemployed	2983 (28.6)	1736 (21.9)
	Both parents unemployed	565 (5.4)	168 (2.1)
	Both parents working	4752 (45.6)	5861 (74.1)
	Missing	2115 (20.3)	149 (1.9)
Mean (SD) number of siblings living in the house at 2 months		0.9 (1)	0.8 (0.9)
Road traffic noise exposure at 2 months	<55 dB(A)	2888 (27.7)	2480 (31.3)
	>=55 dB(A)	3425 (32.9)	2772 (35.0)
	>=60 dB(A)	3355 (32.2)	2662 (33.6)
	Missing	747 (7.2)	0 (0.0)
Road traffic noise exposure at 3.5 years	<55 dB(A)	1012 (9.7)	2428 (30.7)
	>=55 dB(A)	1344 (12.9)	2941 (37.2)
	>=60 dB(A)	1147 (11.0)	2545 (32.2)
	Missing	6912 (66.4)	0 (0.0)
Road traffic noise exposure at 5.5 years	<55 dB(A)	719 (6.9)	2459 (31.1)
	>=55 dB(A)	988 (9.5)	2961 (37.4)
	>=60 dB(A)	803 (7.7)	2494 (31.5)
	Missing	7905 (75.9)	0 (0.0)
Railway traffic noise exposure at 2 months	<50 dB(A)	9491 (91.1)	7753 (98.0)
	>=50 dB(A)	181 (1.7)	161 (2.0)
	Missing	743 (7.1)	0 (0.0)
Railway traffic noise exposure at 3.5 years	<50 dB(A)	3436 (33.0)	7760 (98.1)
	>=50 dB(A)	68 (0.7)	154 (1.9)
	Missing	6911 (66.4)	0 (0.0)
Railway traffic noise exposure at 5.5 years	<50 dB(A)	2510 (24.1)	7914 (100.0)
	Missing	7905 (75.9)	0 (0.0)

Air traffic noise exposure at 2 months	<50 dB(A)	9369 (90.0)	7729 (97.7)
	>=50 dB(A)	303 (2.9)	185 (2.3)
	Missing	743 (7.1)	0 (0.0)
Air traffic noise exposure at 3.5 years	<50 dB(A)	3390 (32.5)	7737 (97.8)
	>=50 dB(A)	114 (1.1)	177 (2.2)
	Missing	6911 (66.4)	0 (0.0)
Air traffic noise exposure at 5.5 years	<50 dB(A)	2438 (23.4)	7723 (97.6)
	>=50 dB(A)	72 (0.7)	191 (2.4)
	Missing	7905 (75.9)	0 (0.0)
Mean (SD) daily screen time at 3.5 years (hours/day)		1.3 (1.1)	1.1 (0.9)
Mean (SD) daily screen time at 5.5 years (hours/day)		1.6 (1.2)	1.3 (1.1)
Residential moving since last time-point at 3.5 years	No	3082 (29.6)	6245 (78.9)
	Yes	826 (7.9)	1534 (19.4)
	Missing	6507 (62.5)	135 (1.7)
Residential moving since last time-point at 5.5 years	No	2497 (24.0)	6226 (78.7)
	Yes	854 (8.2)	1644 (20.8)
	Missing	7064 (67.8)	44 (0.6)
Rural/urban environment at 2 months	< 5,000 inhabitants	2454 (23.6)	2054 (26.0)
	>= 5,000 inhabitants	6086 (58.4)	5793 (73.2)
	Missing	1875 (18.0)	67 (0.8)
Mean (SD) NDVI at 2 months		0.5 (0.2)	0.5 (0.2)
Mean (SD) Particulate Matter 10 at 2 months		23.3 (5.1)	23.2 (4.6)
Mean (SD) SDQ external scale score at 10.5 years		4.9 (3.6)	4.9 (3.7)
Mean (SD) SDQ internal scale score at 10.5 years		4.1 (3.1)	4.1 (3.1)
Mean (SD) SDQ hyperactivity/inattention subscale score at 10.5 years		3.3 (2.6)	3.3 (2.6)
Mean (SD) SDQ conduct problems subscale score at 10.5 years		1.6 (1.6)	1.6 (1.6)
Mean (SD) SDQ emotional problems subscale score at 10.5 years		2.6 (2.2)	2.7 (2.2)
Mean (SD) SDQ peer problems subscale score at 10.5 years		1.5 (1.6)	1.4 (1.5)
SDQ hyperactivity/inattention subscale score at 10.5 years	Normal	1440 (13.8)	6247 (78.9)
	Limit	157 (1.5)	559 (7.1)
	Abnormal	233 (2.2)	1108 (14.0)
	Missing	8585 (82.4)	0 (0.0)
SDQ conduct problems subscale score at 10.5 years	Normal	1374 (13.2)	5956 (75.3)
	Limit	223 (2.1)	987 (12.5)

	Abnormal	233 (2.2)	971 (12.3)
	Missing	8585 (82.4)	0 (0.0)
SDQ emotional problems subscale score at 10.5 years	Normal	1259 (12.1)	5316 (67.2)
	Limit	206 (2.0)	947 (12.0)
	Abnormal	365 (3.5)	1651 (20.9)
	Missing	8585 (82.4)	0 (0.0)
SDQ peer problems subscale score at 10.5 years	Normal	1438 (13.8)	6440 (81.4)
	Limit	197 (1.9)	701 (8.9)
	Abnormal	195 (1.9)	773 (9.8)
	Missing	8585 (82.4)	0 (0.0)

EPDS: Edinburgh Postnatal Depression Scale
NDVI: Normalized Difference Vegetation Index
SD: Standard Deviation
SDQ: Strengths and Difficulties Questionnaire

Values represent numbers (percentages) unless stated otherwise.

Section 3: Modelling of road traffic, aircraft and railway noise

Road traffic noise was modelled using the free and open-source softwares GeoClimate (https://geomanum.org/fr/projets/geoclimate) and NoiseModelling (https://noise-planet.org/noisemodelling.html), relying on open geographic data and a statistical road traffic model derived from the French national AVATAR database (https://avatar.cerema.fr/). Noise emission and propagation was computed using the CNOSSOS-EU method included in NoiseModelling software (described in Commission Directive (EU) 2015/996 of 19 May 2015). Applying an identical numerical workflow to the entire cohort ensured homogeneity, and consistency in the quality of noise estimates and in the associated uncertainties.

Railway noise exposure was derived from official, publicly available strategic noise maps produced by the same UMRAE research team on behalf of the French government. These maps strictly follow the methodology defined for the European strategic noise maps (Directive 2015/996). Only high-traffic railway lines (>30,000 trains per year) were modelled. This standard approach may lead to increased uncertainty in highly urbanised areas, where local conditions such as speed reductions or train station layouts are not fully captured; nevertheless, the same modelling framework was also consistently applied nationwide ensuring consistency.

Aircraft noise exposure was obtained from official, publicly available strategic noise maps, which are also required to comply with the methodology of Directive 2015/996. In this case, noise mapping is conducted by airport authorities, which may result in some variability due to differences in software tools or light methodological choices.

The aim of this stage is to estimate the noise exposure of children in the ELFE cohort. Noise levels were calculated around the building where the child lives. To do this, calculations are made for the building closest to the child postal address, which is assumed to be their home.

The diagram below illustrates this analysis.

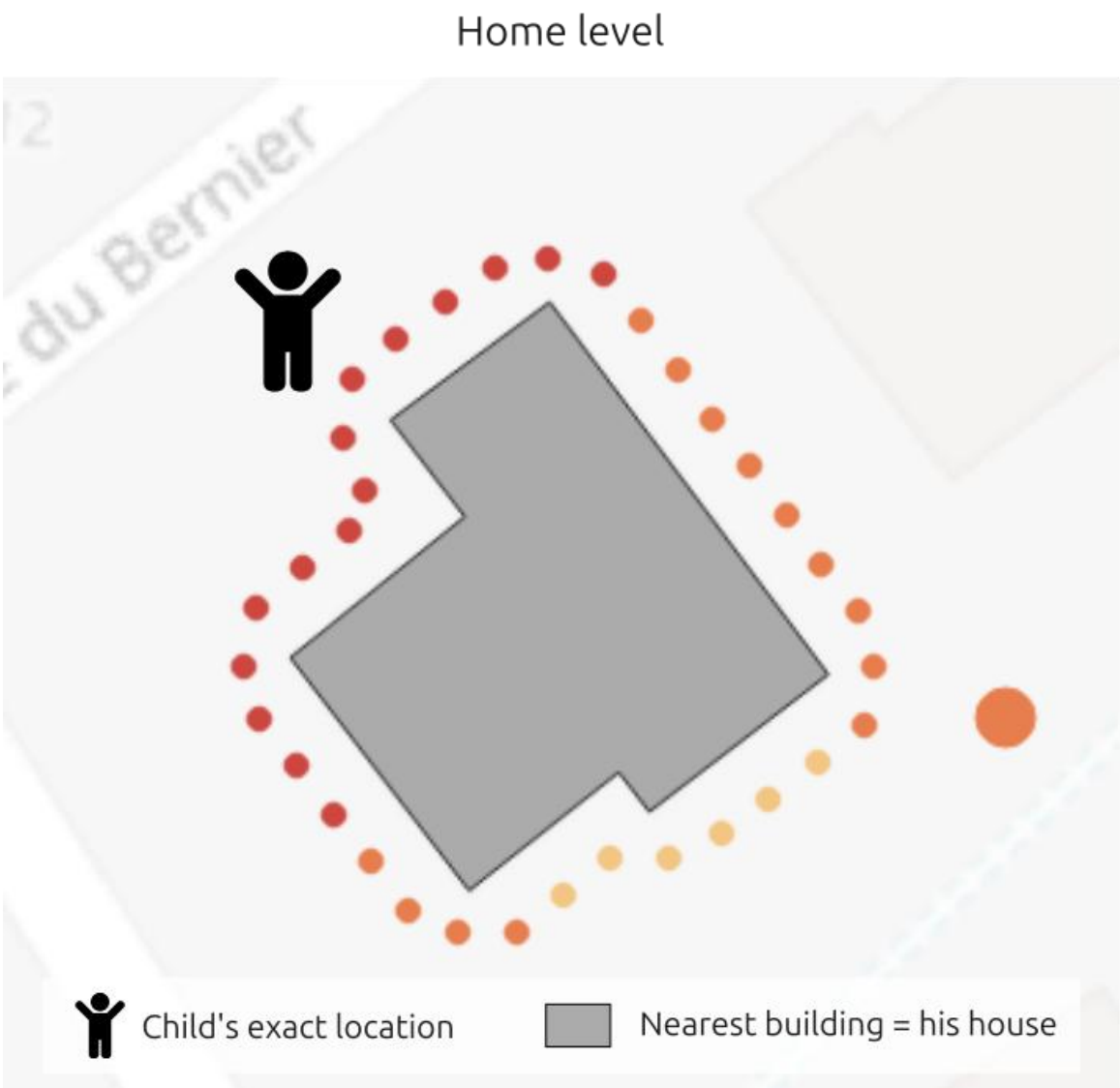

In terms of sound sources, we seek to evaluate:

1. Noise emitted by road traffic, calculated for this purpose using NoiseModelling software and CNOSSOS-EU emission and propagation method.

2. Provided that the receivers created intersect, these sources:
    1. Air traffic noise levels.
    2. Rail traffic noise levels.

Input data

This work is based on the following input datasets:

- **Child registry**: A registry of children, each identified by a unique FID code and geolocated by a pair of planar coordinates (X, Y) expressed in the Lambert 93 projected coordinate reference system (EPSG:2154).
- **Geospatial data**: The OpenStreetMap (OSM) open database, used to extract information on building footprints, land-use classes.
- **Aircraft noise data**: Airborne Noise Exposure Plans (*Plans d'Exposition au Bruit*, PEB) produced by the French Civil Aviation Authority (Direction Générale de l'Aviation Civile, DGAC) and made publicly available via the national geospatial portal: https://geoservices.ign.fr/services-web-experts-transports#2314
- **Railway noise data**: Strategic Noise Maps (*Cartes de Bruit Stratégiques*, CBS) for major railway infrastructures, computed within the PLAMADE project on behalf of the French government (1). Strategic Noise Mapping in France to 2023: Coupling a national database with the open-source model NoiseModelling. In INTER-NOISE and NOISE-CON Congress and Conference Proceedings (Vol. 265, No. 5, pp. 2617-2624). Institute of Noise Control Engineering). In accordance with the European strategic noise mapping framework, only railway lines with annual traffic volumes exceeding 30,000 train passages (approximately 82 trains per day on average) were modelled.
- The CNOSSOS-EU emission and propagation model was parameterized according to the standards used for the production of strategic noise maps, including an 800 m propagation range, first-order reflections, and the consideration of diffraction over horizontal edges, among other settings. Detailed specifications are provided on the platform associated with this publication.

Tools

The processing chain implemented here is based mainly on two free and open-source tools: GeoClimate and NoiseModelling (co)developped by UMRAE team.
- GeoClimate (https://github.com/orbisgis/geoclimate) is used to download and format data from OpenStreetMap, which is then fed into NoiseModelling (2).
- NoiseModelling (https://noise-planet.org/noisemodelling.html) is used to calculate noise levels caused by road traffic at the receivers (house and random) (3).
- The whole process is set to music via a bash script in a Linux (Ubuntu) environment.
- A road traffic estimation package (github.com/Universite-Gustave-Eiffel/OpenDataNoiseMap) which provides a framework for spatiotemporal traffic modeling in France, integrating OpenStreetMap network geometry with existing traffic count data from the CEREMA AVATAR API (https://avatar.cerema.fr/api/doc). The workflow automates data retrieval, network feature engineering with missing attribute imputation, and implements a hierarchical XGBoost modeling strategy. This architecture predicts baseline daytime traffic metrics (flow, speed, heavy vehicle share)

and utilizes learned temporal ratios to derive consistent profiles for specific time periods, ensuring physical validity across vehicle classes. The system enables traffic estimation for road segments and large-scale predictive mapping.

Process

These input datasets are then integrated into the NoiseModelling framework to compute the expected environmental sound levels. At the end of the processing chain, a comprehensive set of acoustic indicators is generated for each child at the residential building receptors. The resulting indicators quantify noise exposure by source and time period and are expressed in A-weighted decibels (dB(A)), reflecting the frequency sensitivity of human hearing.

- for road traffic: L_{DEN} (Days-Evening-Night), L_{DAY} (06-18h), $L_{EVENING}$ (18-22h) and L_{NIGHT} (22-06h)
- for rail traffic: L_{DEN} and L_{NIGHT}, with values discretised in 5dB increments ([55-60[,[60-65[, [65-70[, [70-75 [, >75 for daytime and [50-55[,[55-60[,[60-65[, [65-70[, >70 for night-time)
- for air traffic: L_{DEN}

In an initial phase, noise exposure was computed for all children in the cohort. In a second phase, the calculations were repeated for children who changed residence during the follow-up period, in order to account for variations in noise exposure associated with residential mobility.

All processing was carried out on two moderately powerful laptops and took approximately three weeks.

Section 4: Conceptual DAGs

Environmental covariates include:
- Residential greenness (assessed by NDVI).
- Mean PM10
- Walkability index.
- Rural/urban environment.

For railway and aircraft noise, the DAG reflects a single binary indicator of ever-exposure to ≥50 dB(A) at any time point during follow-up, rather than time-varying exposure. This simplification was necessary due to the low prevalence of exposure to these sources, which precluded stable estimation of time-point-specific causal contrasts.

Arrows from noise exposure to time-varying socioeconomic covariates were not included, reflecting the assumption that residential noise exposure does not materially influence parental employment or income over the study period. While socioeconomic status and residential noise are closely associated at the population level through residential sorting mechanisms, reverse causation within individuals over the follow-up period was considered unlikely.

Supplementary Figure 1: Conceptual DAG of the road traffic noise analyses.

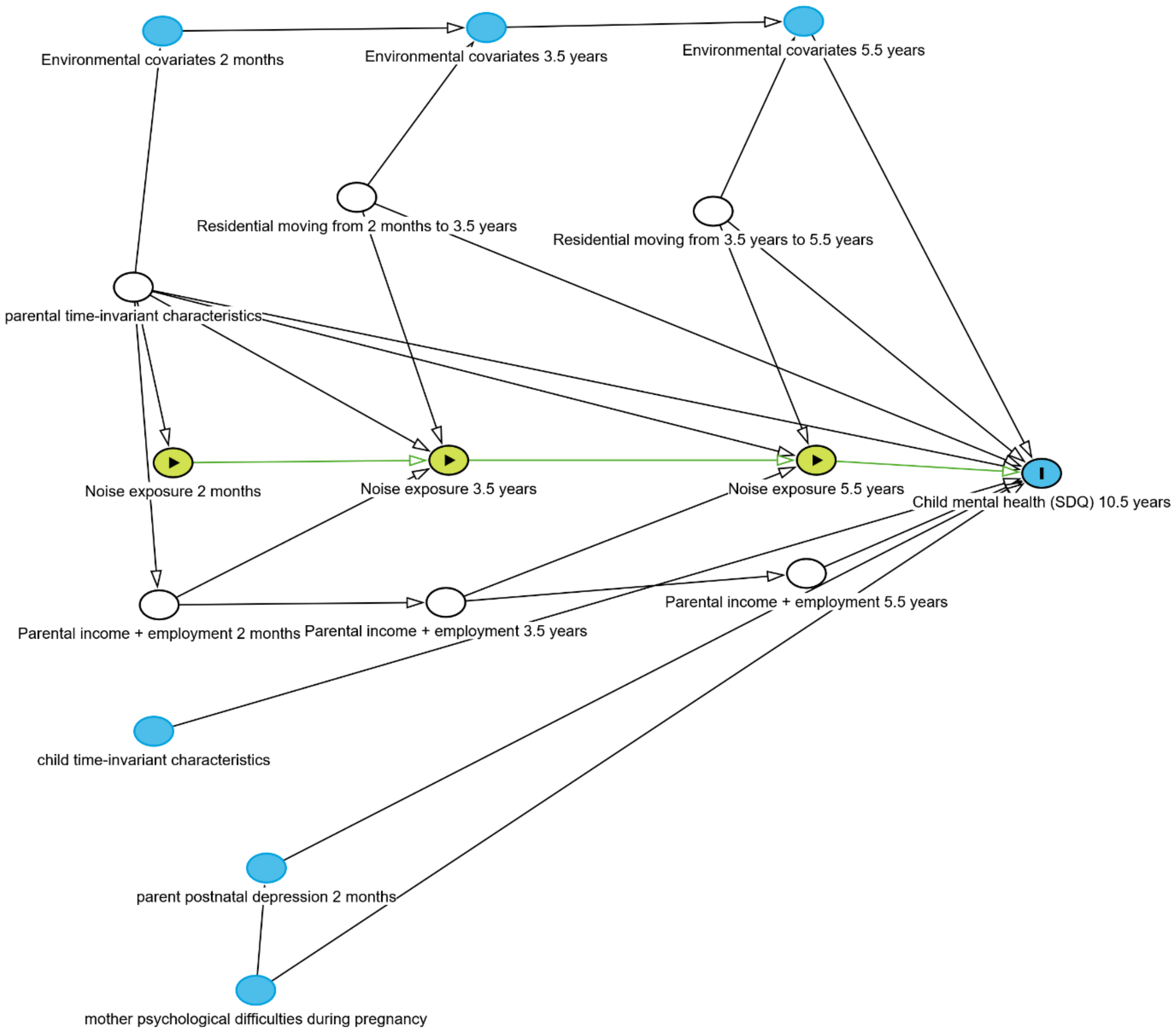

Supplementary Figure 2: Conceptual DAG of the railway and aircraft traffic noise analyses.

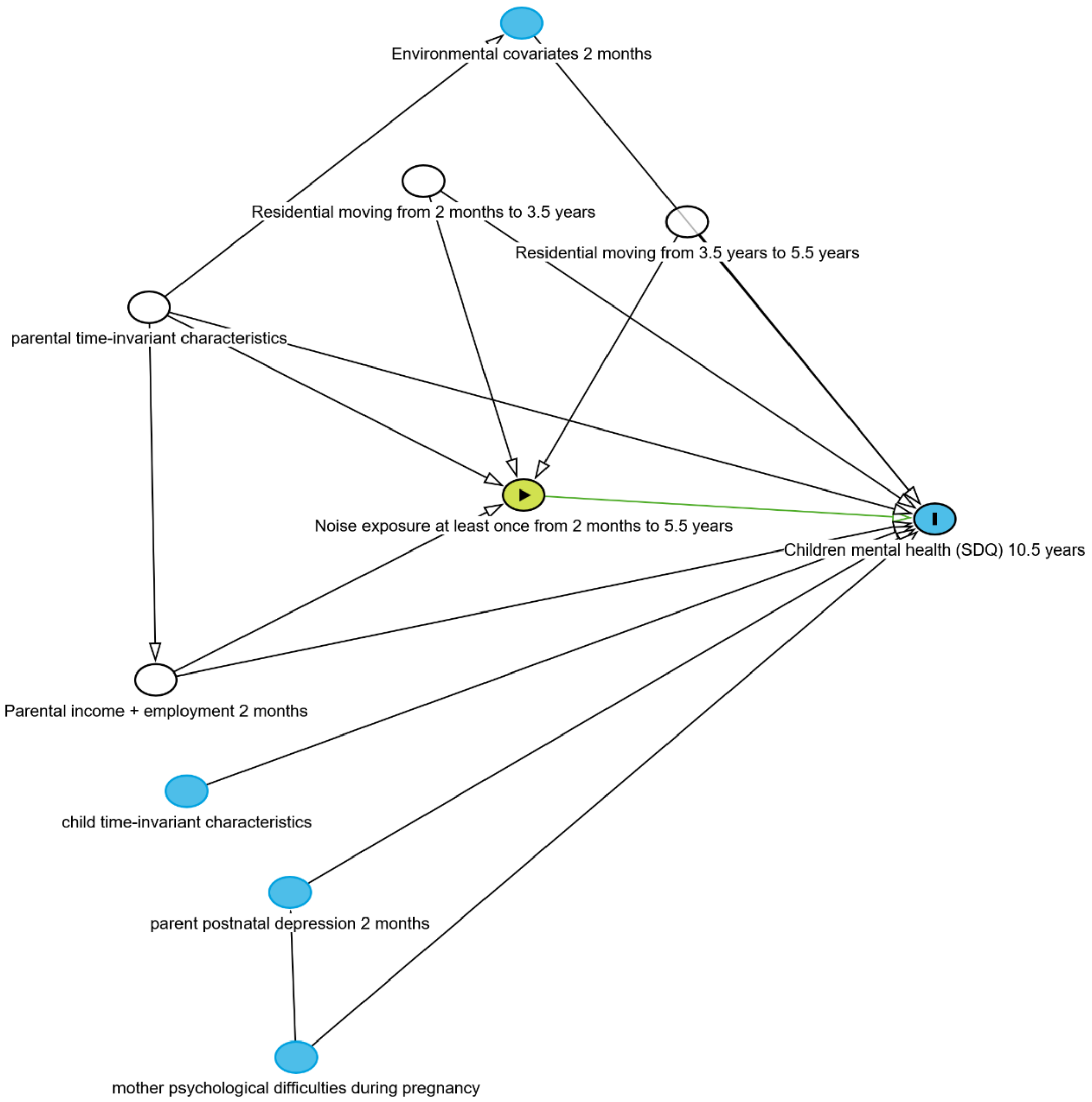

Section 5: Inverse Probability Weights estimation

For estimating the weights for the road traffic noise models, the denominator corresponds to the probability of the observed exposure given the individual's previous exposure status and time-varying confounders. To stabilize the weights, the numerator was defined as the probability of exposure given only the past exposure history and baseline covariates. Because baseline covariates are included in the numerator, they are also included in the final outcome model (4).

For estimating the weights for the railway and aircraft noise models, the denominator corresponded to the probability of the observed exposure given baseline covariates only, as exposure was defined as a binary indicator of being ever exposed during follow-up. To stabilize the weights, the numerator was defined as the probability of exposure given an intercept only (4).

Weights were truncated at the 1st and 99th percentiles to further improve model stability and reduce the effect of extreme weights (4,5). These weights create a pseudo-population in which exposure is independent of measured confounders, allowing unbiased estimation of the marginal causal effect of noise exposure. Weights were estimated using the R package WeightIt.

Absolute standardized mean differences (ASMD) < 0.2 and Kolmogorov-Smirnov (KS) statistic < 0.1 indicated achieved balance for all baseline and time-varying confounders after weighting (6), except for the previous exposure variable at each time point, which was unbalanced. This is expected and reflects the high temporal persistence of residential noise exposure rather than residual confounding (7).

Below is shown covariate balance before and after weighting using absolute standardized mean differences (ASMD) for each covariate, reported as the minimum and maximum values across all imputations. The distribution of IP weights is also shown, indicating minimal presence of extreme weights. This was the case both for main and secondary analyses.

Supplementary Table 2: Road models ASMD report before and after weighting

model	timepoint	variable	modality	asmd_ unweighted_min	asmd_ unweighted_max	asmd_ weighted_min	asmd_ weighted_max
model_road_1	2 months	Sex	2	0,007	0,007	0,008	0,009
model_road_1	2 months	Parental employment at 2 months	1	0,026	0,026	0,006	0,008
model_road_1	2 months	Parental employment at 2 months	2	0,025	0,025	0,006	0,009
model_road_1	2 months	Parental employment at 2 months	3	0,002	0,002	0,000	0,001
model_road_1	2 months	Child's age at questionnaire 10.5 years (in months)		0,034	0,034	0,034	0,036
model_road_1	2 months	Mean (SD) father's age at child's birth		0,014	0,029	0,014	0,033
model_road_1	2 months	Father's migratory status	1	0,002	0,007	0,001	0,007
model_road_1	2 months	Father's migratory status	2	0,011	0,026	0,010	0,026
model_road_1	2 months	Father's migratory status	3	0,014	0,030	0,012	0,029
model_road_1	2 months	Mother's educational level at 2 months	2	0,006	0,007	0,006	0,007
model_road_1	2 months	Mother's educational level at 2 months	3	0,054	0,054	0,055	0,056
model_road_1	2 months	Mother's educational level at 2 months	4	0,030	0,031	0,029	0,031
model_road_1	2 months	Mother's educational level at 2 months	5	0,077	0,078	0,079	0,080
model_road_1	2 months	Mother's migratory status	1	0,011	0,011	0,010	0,011
model_road_1	2 months	Mother's migratory status	2	0,003	0,003	0,002	0,003
model_road_1	2 months	Mother's migratory status	3	0,015	0,015	0,013	0,014
model_road_1	2 months	Mean (SD) mother's age at child's birth		0,057	0,062	0,056	0,063
model_road_1	2 months	Father's educational level at 2 months	2	0,005	0,013	0,005	0,013
model_road_1	2 months	Father's educational level at 2 months	3	0,045	0,059	0,045	0,059
model_road_1	2 months	Father's educational level at 2 months	4	0,024	0,038	0,023	0,037
model_road_1	2 months	Father's educational level at 2 months	5	0,082	0,097	0,081	0,097
model_road_1	2 months	Birth weight		0,001	0,005	0,000	0,005
model_road_1	2 months	Premature birth		0,011	0,016	0,012	0,016
model_road_1	2 months	Income per consumption unit at 2 months (€/month)		0,069	0,078	0,092	0,101
model_road_1	3.5 years	Sex	2	0,007	0,007	0,008	0,009
model_road_1	3.5 years	Parental employment at 2 months	1	0,007	0,007	0,004	0,006
model_road_1	3.5 years	Parental employment at 2 months	2	0,013	0,013	0,006	0,009
model_road_1	3.5 years	Parental employment at 2 months	3	0,007	0,007	0,002	0,003
model_road_1	3.5 years	Parental employment at 3.5 years	1	0,005	0,005	0,005	0,007
model_road_1	3.5 years	Parental employment at 3.5 years	2	0,012	0,012	0,004	0,005
model_road_1	3.5 years	Parental employment at 3.5 years	3	0,007	0,007	0,010	0,011
model_road_1	3.5 years	Child's age at questionnaire 10.5 years (in months)		0,025	0,025	0,027	0,030
model_road_1	3.5 years	Residential moving since last time-point at 3.5 years		0,009	0,015	0,018	0,025
model_road_1	3.5 years	Mean (SD) father's age at child's birth		0,034	0,052	0,027	0,045
model_road_1	3.5 years	Father's migratory status	1	0,003	0,010	0,002	0,008
model_road_1	3.5 years	Father's migratory status	2	0,004	0,021	0,002	0,021
model_road_1	3.5 years	Father's migratory status	3	0,007	0,026	0,006	0,024
model_road_1	3.5 years	Mother's educational level at 2 months	2	0,006	0,006	0,007	0,008
model_road_1	3.5 years	Mother's educational level at 2 months	3	0,044	0,045	0,045	0,046
model_road_1	3.5 years	Mother's educational level at 2 months	4	0,029	0,030	0,028	0,029
model_road_1	3.5 years	Mother's educational level at 2 months	5	0,070	0,071	0,070	0,071
model_road_1	3.5 years	Mother's migratory status	1	0,013	0,013	0,012	0,012
model_road_1	3.5 years	Mother's migratory status	2	0,002	0,002	0,002	0,002

model_road_1	3.5 years	Mother's migratory status	3	0,015	0,015	0,013	0,014
model_road_1	3.5 years	Mean (SD) mother's age at child's birth		0,072	0,078	0,067	0,072
model_road_1	3.5 years	Father's educational level at 2 months	2	0,015	0,026	0,016	0,027
model_road_1	3.5 years	Father's educational level at 2 months	3	0,039	0,058	0,038	0,058
model_road_1	3.5 years	Father's educational level at 2 months	4	0,027	0,039	0,027	0,039
model_road_1	3.5 years	Father's educational level at 2 months	5	0,079	0,094	0,078	0,093
model_road_1	3.5 years	Birth weight		0,003	0,006	0,003	0,006
model_road_1	3.5 years	Premature birth		0,005	0,010	0,006	0,011
model_road_1	3.5 years	Income per consumption unit at 2 months (€/month)		0,095	0,108	0,087	0,097
model_road_1	3.5 years	Income per consumption unit at 3.5 years (€/month)		0,123	0,149	0,088	0,105
model_road_1	3.5 years	Road traffic noise exposure at 2 months		0,629	0,629	0,627	0,628
model_road_1	3.5 years	Road traffic noise exposure at 2 months		0,645	0,645	0,644	0,646
model_road_1	3.5 years	Road traffic noise exposure at 2 months		0,650	0,650	0,645	0,647
model_road_1	5.5 years	Sex	2	0,014	0,014	0,012	0,013
model_road_1	5.5 years	Parental employment at 2 months	1	0,015	0,015	0,001	0,003
model_road_1	5.5 years	Parental employment at 2 months	2	0,022	0,022	0,004	0,006
model_road_1	5.5 years	Parental employment at 2 months	3	0,010	0,010	0,003	0,004
model_road_1	5.5 years	Parental employment at 3.5 years	1	0,007	0,007	0,004	0,006
model_road_1	5.5 years	Parental employment at 3.5 years	2	0,006	0,006	0,004	0,006
model_road_1	5.5 years	Parental employment at 3.5 years	3	0,014	0,014	0,005	0,007
model_road_1	5.5 years	Parental employment at 5.5 years	1	0,016	0,016	0,015	0,016
model_road_1	5.5 years	Parental employment at 5.5 years	2	0,013	0,013	0,019	0,021
model_road_1	5.5 years	Parental employment at 5.5 years	3	0,007	0,007	0,008	0,009
model_road_1	5.5 years	Child's age at questionnaire 10.5 years (in months)		0,011	0,011	0,009	0,012
model_road_1	5.5 years	Residential moving since last time-point at 3.5 years		0,002	0,013	0,012	0,018
model_road_1	5.5 years	Residential moving since last time-point at 5.5 years		0,019	0,025	0,017	0,021
model_road_1	5.5 years	Mean (SD) father's age at child's birth		0,031	0,047	0,025	0,041
model_road_1	5.5 years	Father's migratory status	1	0,002	0,008	0,003	0,009
model_road_1	5.5 years	Father's migratory status	2	0,001	0,014	0,001	0,012
model_road_1	5.5 years	Father's migratory status	3	0,000	0,013	0,001	0,013
model_road_1	5.5 years	Mother's educational level at 2 months	2	0,002	0,002	0,002	0,003
model_road_1	5.5 years	Mother's educational level at 2 months	3	0,052	0,052	0,050	0,051
model_road_1	5.5 years	Mother's educational level at 2 months	4	0,026	0,026	0,026	0,027
model_road_1	5.5 years	Mother's educational level at 2 months	5	0,077	0,078	0,073	0,074
model_road_1	5.5 years	Mother's migratory status	1	0,015	0,015	0,015	0,016
model_road_1	5.5 years	Mother's migratory status	2	0,002	0,002	0,001	0,001
model_road_1	5.5 years	Mother's migratory status	3	0,016	0,016	0,015	0,016
model_road_1	5.5 years	Mean (SD) mother's age at child's birth		0,062	0,068	0,055	0,061
model_road_1	5.5 years	Father's educational level at 2 months	2	0,010	0,021	0,009	0,021
model_road_1	5.5 years	Father's educational level at 2 months	3	0,039	0,056	0,039	0,056
model_road_1	5.5 years	Father's educational level at 2 months	4	0,017	0,027	0,018	0,028
model_road_1	5.5 years	Father's educational level at 2 months	5	0,080	0,092	0,078	0,090
model_road_1	5.5 years	Birth weight		0,005	0,008	0,005	0,008
model_road_1	5.5 years	Premature birth		0,013	0,018	0,012	0,018
model_road_1	5.5 years	Income per consumption unit at 2 months (€/month)		0,128	0,141	0,091	0,102
model_road_1	5.5 years	Income per consumption unit at 3.5 years (€/month)		0,141	0,164	0,095	0,108

model_road_1	5.5 years	Income per consumption unit at 5.5 years (€/month)		0,120	0,141	0,074	0,089
model_road_1	5.5 years	Road traffic noise exposure at 2 months		0,493	0,493	0,492	0,494
model_road_1	5.5 years	Road traffic noise exposure at 2 months		0,530	0,530	0,529	0,531
model_road_1	5.5 years	Road traffic noise exposure at 2 months		0,525	0,525	0,523	0,525
model_road_1	5.5 years	Road traffic noise exposure at 3.5 years		0,773	0,773	0,775	0,777
model_road_1	5.5 years	Road traffic noise exposure at 3.5 years		0,799	0,799	0,795	0,797
model_road_1	5.5 years	Road traffic noise exposure at 3.5 years		0,799	0,799	0,796	0,799
model_road_2	2 months	Sex	2	0,007	0,007	0,006	0,009
model_road_2	2 months	Parental employment at 2 months	1	0,026	0,026	0,010	0,013
model_road_2	2 months	Parental employment at 2 months	2	0,025	0,025	0,010	0,014
model_road_2	2 months	Parental employment at 2 months	3	0,002	0,002	0,002	0,003
model_road_2	2 months	Child's age at questionnaire 10.5 years (in months)		0,034	0,034	0,041	0,047
model_road_2	2 months	Mean (SD) father's age at child's birth		0,014	0,029	0,036	0,060
model_road_2	2 months	Father's migratory status	1	0,002	0,007	0,002	0,008
model_road_2	2 months	Father's migratory status	2	0,011	0,026	0,011	0,029
model_road_2	2 months	Father's migratory status	3	0,014	0,030	0,013	0,032
model_road_2	2 months	Mean (SD) Particulate Matter 10 at 2 months		0,387	0,406	0,058	0,068
model_road_2	2 months	Mother's educational level at 2 months	2	0,006	0,007	0,008	0,010
model_road_2	2 months	Mother's educational level at 2 months	3	0,054	0,054	0,060	0,062
model_road_2	2 months	Mother's educational level at 2 months	4	0,030	0,031	0,027	0,029
model_road_2	2 months	Mother's educational level at 2 months	5	0,077	0,078	0,080	0,084
model_road_2	2 months	Mother's migratory status	1	0,011	0,011	0,014	0,016
model_road_2	2 months	Mother's migratory status	2	0,003	0,003	0,006	0,008
model_road_2	2 months	Mother's migratory status	3	0,015	0,015	0,020	0,024
model_road_2	2 months	Mean (SD) mother's age at child's birth		0,057	0,062	0,073	0,080
model_road_2	2 months	Mother psychological difficulties during pregnancy		0,004	0,009	0,006	0,009
model_road_2	2 months	Father's educational level at 2 months	2	0,005	0,013	0,008	0,018
model_road_2	2 months	Father's educational level at 2 months	3	0,045	0,059	0,052	0,065
model_road_2	2 months	Father's educational level at 2 months	4	0,024	0,038	0,020	0,033
model_road_2	2 months	Father's educational level at 2 months	5	0,082	0,097	0,088	0,105
model_road_2	2 months	Mean (SD) number of siblings living in the house at 2 months		0,045	0,045	0,013	0,019
model_road_2	2 months	Mean (SD) NDVI at 2 months		0,526	0,536	0,060	0,068
model_road_2	2 months	Parental postnatal depression risk (EPDS scale)		0,002	0,014	0,002	0,015
model_road_2	2 months	Birth weight		0,001	0,005	0,001	0,004
model_road_2	2 months	Premature birth		0,011	0,016	0,014	0,018
model_road_2	2 months	Income per consumption unit at 2 months (€/month)		0,069	0,078	0,091	0,103
model_road_2	2 months	Walkability index at 2 months (tertiles)		0,236	0,244	0,047	0,055
model_road_2	3.5 years	Sex	2	0,007	0,007	0,017	0,020
model_road_2	3.5 years	Mean (SD) daily screen time at 3.5 years (hours/day)		0,117	0,125	0,086	0,094
model_road_2	3.5 years	Parental employment at 2 months	1	0,007	0,007	0,010	0,014
model_road_2	3.5 years	Parental employment at 2 months	2	0,013	0,013	0,013	0,016
model_road_2	3.5 years	Parental employment at 2 months	3	0,007	0,007	0,003	0,005
model_road_2	3.5 years	Parental employment at 3.5 years	1	0,005	0,005	0,007	0,009
model_road_2	3.5 years	Parental employment at 3.5 years	2	0,012	0,012	0,006	0,010
model_road_2	3.5 years	Parental employment at 3.5 years	3	0,007	0,007	0,010	0,012
model_road_2	3.5 years	Child's age at questionnaire 10.5 years (in months)		0,025	0,025	0,029	0,036

model_road_2	3.5 years	Residential moving since last time-point at 3.5 years		0,009	0,015	0,011	0,019
model_road_2	3.5 years	Mean (SD) father's age at child's birth		0,034	0,052	0,056	0,070
model_road_2	3.5 years	Father's migratory status	1	0,003	0,010	0,002	0,009
model_road_2	3.5 years	Father's migratory status	2	0,004	0,021	0,003	0,018
model_road_2	3.5 years	Father's migratory status	3	0,007	0,026	0,002	0,016
model_road_2	3.5 years	Mean (SD) Particulate Matter 10 at 2 months		0,336	0,356	0,058	0,069
model_road_2	3.5 years	Mean (SD) Particulate Matter 10 at 3.5 years		0,395	0,419	0,076	0,091
model_road_2	3.5 years	Mother's educational level at 2 months	2	0,006	0,006	0,005	0,006
model_road_2	3.5 years	Mother's educational level at 2 months	3	0,044	0,045	0,049	0,052
model_road_2	3.5 years	Mother's educational level at 2 months	4	0,029	0,030	0,029	0,031
model_road_2	3.5 years	Mother's educational level at 2 months	5	0,070	0,071	0,075	0,078
model_road_2	3.5 years	Mother's migratory status	1	0,013	0,013	0,005	0,007
model_road_2	3.5 years	Mother's migratory status	2	0,002	0,002	0,006	0,008
model_road_2	3.5 years	Mother's migratory status	3	0,015	0,015	0,010	0,013
model_road_2	3.5 years	Mean (SD) mother's age at child's birth		0,072	0,078	0,100	0,108
model_road_2	3.5 years	Mother psychological difficulties during pregnancy		0,011	0,016	0,015	0,019
model_road_2	3.5 years	Father's educational level at 2 months	2	0,015	0,026	0,019	0,031
model_road_2	3.5 years	Father's educational level at 2 months	3	0,039	0,058	0,043	0,064
model_road_2	3.5 years	Father's educational level at 2 months	4	0,027	0,039	0,031	0,042
model_road_2	3.5 years	Father's educational level at 2 months	5	0,079	0,094	0,087	0,102
model_road_2	3.5 years	Mean (SD) number of siblings living in the house at 2 months		0,029	0,029	0,011	0,016
model_road_2	3.5 years	Mean (SD) number of siblings living in the house at 3.5 years		0,042	0,047	0,034	0,042
model_road_2	3.5 years	Mean (SD) NDVI at 2 months		0,399	0,410	0,054	0,062
model_road_2	3.5 years	Mean (SD) NDVI at 3.5 years		0,473	0,485	0,064	0,076
model_road_2	3.5 years	Parental postnatal depression risk (EPDS scale)		0,006	0,020	0,006	0,025
model_road_2	3.5 years	Birth weight		0,003	0,006	0,004	0,008
model_road_2	3.5 years	Premature birth		0,005	0,010	0,009	0,014
model_road_2	3.5 years	Income per consumption unit at 2 months (€/month)		0,095	0,108	0,101	0,113
model_road_2	3.5 years	Income per consumption unit at 3.5 years (€/month)		0,123	0,149	0,108	0,127
model_road_2	3.5 years	Road traffic noise exposure at 2 months		0,629	0,629	0,623	0,627
model_road_2	3.5 years	Road traffic noise exposure at 2 months		0,645	0,645	0,629	0,633
model_road_2	3.5 years	Road traffic noise exposure at 2 months		0,650	0,650	0,640	0,643
model_road_2	3.5 years	Walkability index at 2 months (tertiles)		0,197	0,204	0,034	0,040
model_road_2	3.5 years	Walkability index at 3.5 years (tertiles)		0,211	0,217	0,016	0,024
model_road_2	5.5 years	Sex	2	0,014	0,014	0,008	0,011
model_road_2	5.5 years	Mean (SD) daily screen time at 3.5 years (hours/day)		0,111	0,120	0,083	0,092
model_road_2	5.5 years	Mean (SD) daily screen time at 5.5 years (hours/day)		0,085	0,089	0,064	0,070
model_road_2	5.5 years	Parental employment at 2 months	1	0,015	0,015	0,005	0,010
model_road_2	5.5 years	Parental employment at 2 months	2	0,022	0,022	0,007	0,011
model_road_2	5.5 years	Parental employment at 2 months	3	0,010	0,010	0,003	0,005
model_road_2	5.5 years	Parental employment at 3.5 years	1	0,007	0,007	0,002	0,005
model_road_2	5.5 years	Parental employment at 3.5 years	2	0,006	0,006	0,007	0,009
model_road_2	5.5 years	Parental employment at 3.5 years	3	0,014	0,014	0,006	0,009
model_road_2	5.5 years	Parental employment at 5.5 years	1	0,016	0,016	0,016	0,018
model_road_2	5.5 years	Parental employment at 5.5 years	2	0,013	0,013	0,026	0,029
model_road_2	5.5 years	Parental employment at 5.5 years	3	0,007	0,007	0,010	0,012

model_road_2	5.5 years	Child's age at questionnaire 10.5 years (in months)		0,011	0,011	0,031	0,039
model_road_2	5.5 years	Residential moving since last time-point at 3.5 years		0,002	0,013	0,007	0,013
model_road_2	5.5 years	Residential moving since last time-point at 5.5 years		0,019	0,025	0,009	0,014
model_road_2	5.5 years	Mean (SD) father's age at child's birth		0,031	0,047	0,015	0,033
model_road_2	5.5 years	Father's migratory status	1	0,002	0,008	0,002	0,009
model_road_2	5.5 years	Father's migratory status	2	0,001	0,014	0,002	0,015
model_road_2	5.5 years	Father's migratory status	3	0,000	0,013	0,002	0,018
model_road_2	5.5 years	Mean (SD) Particulate Matter 10 at 2 months		0,308	0,324	0,059	0,066
model_road_2	5.5 years	Mean (SD) Particulate Matter 10 at 3.5 years		0,359	0,375	0,080	0,092
model_road_2	5.5 years	Mean (SD) Particulate Matter 10 at 5.5 years		0,302	0,319	0,057	0,071
model_road_2	5.5 years	Mother's educational level at 2 months	2	0,002	0,002	0,001	0,003
model_road_2	5.5 years	Mother's educational level at 2 months	3	0,052	0,052	0,059	0,062
model_road_2	5.5 years	Mother's educational level at 2 months	4	0,026	0,026	0,024	0,027
model_road_2	5.5 years	Mother's educational level at 2 months	5	0,077	0,078	0,082	0,084
model_road_2	5.5 years	Mother's migratory status	1	0,015	0,015	0,009	0,012
model_road_2	5.5 years	Mother's migratory status	2	0,002	0,002	0,001	0,003
model_road_2	5.5 years	Mother's migratory status	3	0,016	0,016	0,011	0,014
model_road_2	5.5 years	Mean (SD) mother's age at child's birth		0,062	0,068	0,057	0,065
model_road_2	5.5 years	Mother psychological difficulties during pregnancy		0,023	0,028	0,026	0,031
model_road_2	5.5 years	Father's educational level at 2 months	2	0,010	0,021	0,011	0,026
model_road_2	5.5 years	Father's educational level at 2 months	3	0,039	0,056	0,042	0,065
model_road_2	5.5 years	Father's educational level at 2 months	4	0,017	0,027	0,028	0,037
model_road_2	5.5 years	Father's educational level at 2 months	5	0,080	0,092	0,095	0,107
model_road_2	5.5 years	Mean (SD) number of siblings living in the house at 2 months		0,029	0,029	0,039	0,047
model_road_2	5.5 years	Mean (SD) number of siblings living in the house at 3.5 years		0,039	0,045	0,006	0,016
model_road_2	5.5 years	Mean (SD) number of siblings living in the house at 5.5 years		0,032	0,034	0,014	0,022
model_road_2	5.5 years	Mean (SD) NDVI at 2 months		0,322	0,334	0,090	0,097
model_road_2	5.5 years	Mean (SD) NDVI at 3.5 years		0,389	0,402	0,041	0,048
model_road_2	5.5 years	Mean (SD) NDVI at 5.5 years		0,429	0,443	0,061	0,071
model_road_2	5.5 years	Parental postnatal depression risk (EPDS scale)		0,004	0,016	0,003	0,016
model_road_2	5.5 years	Birth weight		0,005	0,008	0,006	0,009
model_road_2	5.5 years	Premature birth		0,013	0,018	0,014	0,019
model_road_2	5.5 years	Income per consumption unit at 2 months (€/month)		0,128	0,141	0,100	0,111
model_road_2	5.5 years	Income per consumption unit at 3.5 years (€/month)		0,141	0,164	0,100	0,117
model_road_2	5.5 years	Income per consumption unit at 5.5 years (€/month)		0,120	0,141	0,077	0,096
model_road_2	5.5 years	Road traffic noise exposure at 2 months		0,493	0,493	0,485	0,488
model_road_2	5.5 years	Road traffic noise exposure at 2 months		0,530	0,530	0,508	0,512
model_road_2	5.5 years	Road traffic noise exposure at 2 months		0,525	0,525	0,517	0,519
model_road_2	5.5 years	Road traffic noise exposure at 3.5 years		0,773	0,773	0,771	0,774
model_road_2	5.5 years	Road traffic noise exposure at 3.5 years		0,799	0,799	0,781	0,785
model_road_2	5.5 years	Road traffic noise exposure at 3.5 years		0,799	0,799	0,788	0,791
model_road_2	5.5 years	Walkability index at 2 months (tertiles)		0,164	0,171	0,045	0,054
model_road_2	5.5 years	Walkability index at 3.5 years (tertiles)		0,178	0,183	0,023	0,028
model_road_2	5.5 years	Walkability index at 5.5 years (tertiles)		0,163	0,164	0,022	0,030

ASMD = Absolute Standardized Mean Difference.
ASMD before and after weighting using absolute standardized mean differences for each covariate, reported as the minimum and maximum values across all imputations.
Imbalance of previous exposure variable is expected and reflects the high temporal persistence of residential noise exposure rather than residual confounding.

Supplementary Figure 3: Histogram road model 2 (mean of weights= 1.012):

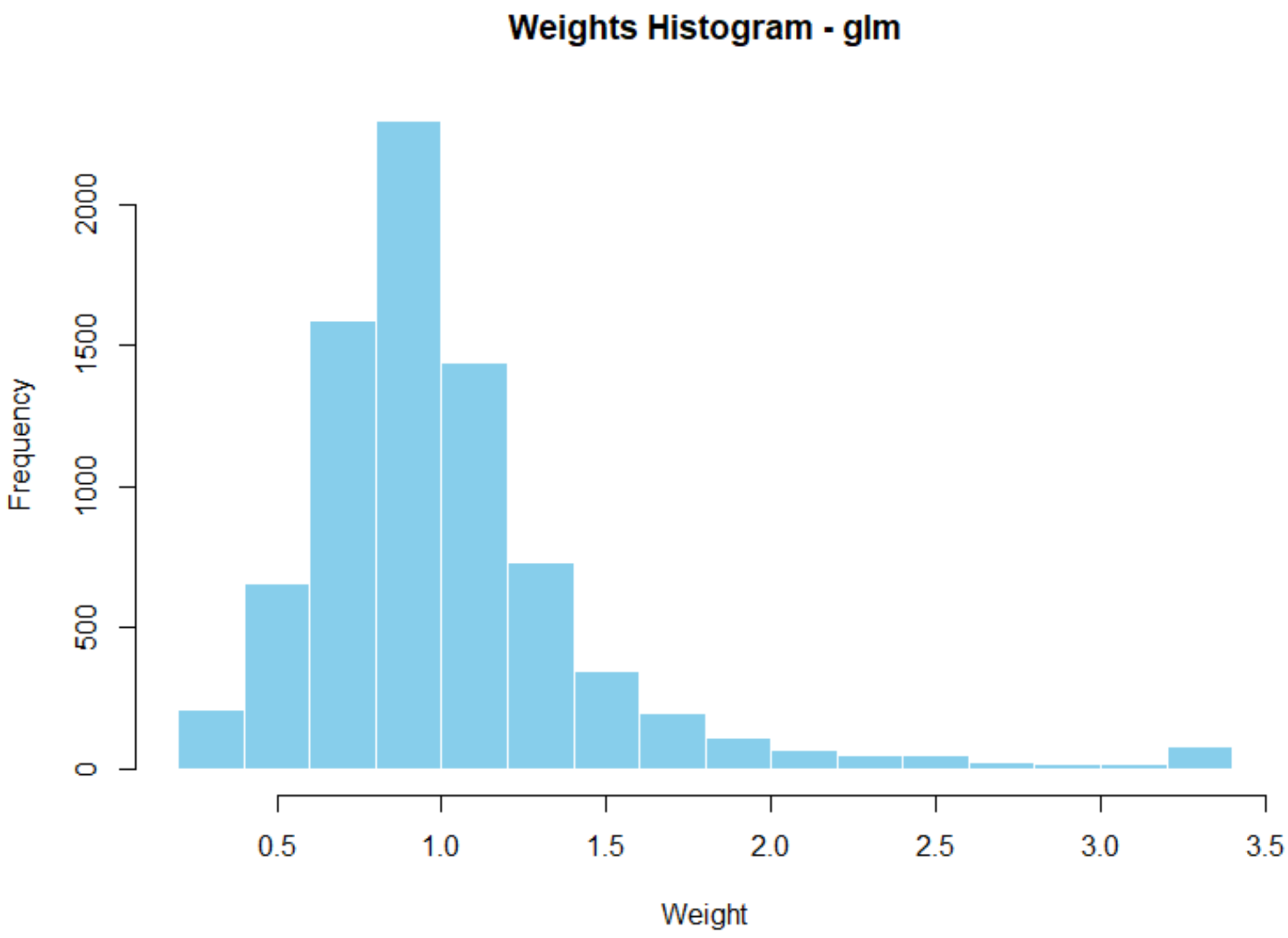

Supplementary Table 3: Aircraft and railway models ASMD reports before and after weighting

model	covariate	modality	asmd unweighted min	asmd unweighted max	asmd weighted min	asmd weighted max
model_aero_1	Sex	2	0,002	0,002	0,000	0,007
model_aero_1	Parental employment at 2 months	1	0,031	0,031	0,002	0,009
model_aero_1	Parental employment at 2 months	2	0,052	0,052	0,015	0,022
model_aero_1	Parental employment at 2 months	3	0,021	0,021	0,010	0,015
model_aero_1	Child's age at questionnaire 10.5 years (in months)		0,090	0,090	0,051	0,065
model_aero_1	Residential moving since last time-point at 3.5 years		0,085	0,113	0,044	0,054
model_aero_1	Residential moving since last time-point at 5.5 years		0,099	0,108	0,062	0,070
model_aero_1	Mean (SD) father's age at child's birth		0,120	0,146	0,054	0,081
model_aero_1	Father's migratory status	1	0,029	0,045	0,012	0,022
model_aero_1	Father's migratory status	2	0,073	0,105	0,028	0,047
model_aero_1	Father's migratory status	3	0,107	0,144	0,044	0,062
model_aero_1	Mother's educational level at 2 months	2	0,031	0,043	0,016	0,023
model_aero_1	Mother's educational level at 2 months	3	0,000	0,007	0,002	0,009
model_aero_1	Mother's educational level at 2 months	4	0,037	0,042	0,020	0,026
model_aero_1	Mother's educational level at 2 months	5	0,004	0,012	0,008	0,013
model_aero_1	Mother's migratory status	1	0,093	0,108	0,037	0,046
model_aero_1	Mother's migratory status	2	0,065	0,073	0,022	0,029
model_aero_1	Mother's migratory status	3	0,163	0,173	0,059	0,073
model_aero_1	Mean (SD) mother's age at child's birth		0,148	0,159	0,084	0,098
model_aero_1	Father's educational level at 2 months	2	0,019	0,051	0,017	0,030
model_aero_1	Father's educational level at 2 months	3	0,013	0,061	0,010	0,032
model_aero_1	Father's educational level at 2 months	4	0,004	0,039	0,011	0,030
model_aero_1	Father's educational level at 2 months	5	0,009	0,042	0,010	0,031
model_aero_1	Birth weight		0,002	0,011	0,002	0,010
model_aero_1	Premature birth		0,003	0,011	0,000	0,003
model_aero_1	Income per consumption unit at 2 months (€/month)		0,003	0,040	0,000	0,017
model_aero_2	Sex	2	0,002	0,002	0,006	0,011
model_aero_2	Mean (SD) daily screen time at 3.5 years (hours/day)		0,001	0,024	0,032	0,051
model_aero_2	Mean (SD) daily screen time at 5.5 years (hours/day)		0,038	0,038	0,000	0,012
model_aero_2	Parental employment at 2 months	1	0,031	0,031	0,006	0,013
model_aero_2	Parental employment at 2 months	2	0,052	0,052	0,017	0,025
model_aero_2	Parental employment at 2 months	3	0,021	0,021	0,008	0,015
model_aero_2	Child's age at questionnaire 10.5 years (in months)		0,090	0,090	0,036	0,051
model_aero_2	Residential moving since last time-point at 3.5 years		0,085	0,113	0,045	0,054
model_aero_2	Residential moving since last time-point at 5.5 years		0,099	0,108	0,058	0,067
model_aero_2	Mean (SD) father's age at child's birth		0,120	0,146	0,028	0,058
model_aero_2	Father's migratory status	1	0,029	0,045	0,013	0,021
model_aero_2	Father's migratory status	2	0,073	0,105	0,041	0,057
model_aero_2	Father's migratory status	3	0,107	0,144	0,055	0,073
model_aero_2	Mean (SD) Particulate Matter 10 at 2 months		0,345	0,373	0,190	0,203
model_aero_2	Mother's educational level at 2 months	2	0,031	0,043	0,016	0,023
model_aero_2	Mother's educational level at 2 months	3	0,000	0,007	0,007	0,015
model_aero_2	Mother's educational level at 2 months	4	0,037	0,042	0,030	0,038

model_aero_2	Mother's educational level at 2 months	5	0,004	0,012	0,022	0,029
model_aero_2	Mother's migratory status	1	0,093	0,108	0,044	0,051
model_aero_2	Mother's migratory status	2	0,065	0,073	0,027	0,033
model_aero_2	Mother's migratory status	3	0,163	0,173	0,074	0,082
model_aero_2	Mean (SD) mother's age at child's birth		0,148	0,159	0,048	0,071
model_aero_2	Mother psychological difficulties during pregnancy		0,002	0,010	0,000	0,006
model_aero_2	Father's educational level at 2 months	2	0,019	0,051	0,019	0,034
model_aero_2	Father's educational level at 2 months	3	0,013	0,061	0,024	0,041
model_aero_2	Father's educational level at 2 months	4	0,004	0,039	0,016	0,032
model_aero_2	Father's educational level at 2 months	5	0,009	0,042	0,025	0,043
model_aero_2	Mean (SD) number of siblings living in the house at 2 months		0,055	0,072	0,033	0,049
model_aero_2	Mean (SD) NDVI at 2 months		0,287	0,338	0,129	0,198
model_aero_2	Parental postnatal depression risk (EPDS scale)		0,001	0,036	0,000	0,007
model_aero_2	Birth weight		0,002	0,011	0,001	0,009
model_aero_2	Premature birth		0,003	0,011	0,000	0,006
model_aero_2	Income per consumption unit at 2 months (€/month)		0,003	0,040	0,005	0,032
model_aero_2	Rural/urban environment at 2 months		0,199	0,203	0,162	0,169
model_aero_2	Walkability index at 2 months (tertiles)		0,214	0,239	0,158	0,179
model_ferro_1	Sex	2	0,001	0,001	0,010	0,017
model_ferro_1	Parental employment at 2 months	1	0,009	0,009	0,001	0,006
model_ferro_1	Parental employment at 2 months	2	0,005	0,005	0,000	0,004
model_ferro_1	Parental employment at 2 months	3	0,004	0,004	0,000	0,005
model_ferro_1	Child's age at questionnaire 10.5 years (in months)		0,149	0,149	0,087	0,103
model_ferro_1	Residential moving since last time-point at 3.5 years		0,088	0,108	0,045	0,062
model_ferro_1	Residential moving since last time-point at 5.5 years		0,064	0,079	0,034	0,043
model_ferro_1	Mean (SD) father's age at child's birth		0,045	0,077	0,002	0,030
model_ferro_1	Father's migratory status	1	0,022	0,037	0,013	0,026
model_ferro_1	Father's migratory status	2	0,043	0,081	0,029	0,051
model_ferro_1	Father's migratory status	3	0,075	0,108	0,047	0,069
model_ferro_1	Mother's educational level at 2 months	2	0,016	0,022	0,014	0,020
model_ferro_1	Mother's educational level at 2 months	3	0,069	0,075	0,045	0,052
model_ferro_1	Mother's educational level at 2 months	4	0,029	0,035	0,014	0,021
model_ferro_1	Mother's educational level at 2 months	5	0,014	0,020	0,008	0,014
model_ferro_1	Mother's migratory status	1	0,046	0,052	0,029	0,033
model_ferro_1	Mother's migratory status	2	0,000	0,006	0,000	0,003
model_ferro_1	Mother's migratory status	3	0,047	0,053	0,029	0,034
model_ferro_1	Mean (SD) mother's age at child's birth		0,105	0,119	0,062	0,076
model_ferro_1	Father's educational level at 2 months	2	0,007	0,035	0,006	0,024
model_ferro_1	Father's educational level at 2 months	3	0,027	0,074	0,023	0,050
model_ferro_1	Father's educational level at 2 months	4	0,016	0,049	0,021	0,040
model_ferro_1	Father's educational level at 2 months	5	0,019	0,061	0,010	0,037
model_ferro_1	Birth weight		0,010	0,015	0,005	0,009
model_ferro_1	Premature birth		0,007	0,013	0,000	0,005
model_ferro_1	Income per consumption unit at 2 months (€/month)		0,020	0,052	0,003	0,032
model_ferro_2	Sex	2	0,001	0,001	0,009	0,018
model_ferro_2	Mean (SD) daily screen time at 3.5 years (hours/day)		0,079	0,105	0,059	0,085

model_ferro_2	Mean (SD) daily screen time at 5.5 years (hours/day)		0,091	0,091	0,023	0,038
model_ferro_2	Parental employment at 2 months	1	0,009	0,009	0,003	0,012
model_ferro_2	Parental employment at 2 months	2	0,005	0,005	0,003	0,011
model_ferro_2	Parental employment at 2 months	3	0,004	0,004	0,000	0,003
model_ferro_2	Child's age at questionnaire 10.5 years (in months)		0,149	0,149	0,071	0,091
model_ferro_2	Residential moving since last time-point at 3.5 years		0,088	0,108	0,049	0,065
model_ferro_2	Residential moving since last time-point at 5.5 years		0,064	0,079	0,038	0,049
model_ferro_2	Mean (SD) father's age at child's birth		0,045	0,077	0,013	0,038
model_ferro_2	Father's migratory status	1	0,022	0,037	0,011	0,021
model_ferro_2	Father's migratory status	2	0,043	0,081	0,028	0,051
model_ferro_2	Father's migratory status	3	0,075	0,108	0,046	0,066
model_ferro_2	Mean (SD) Particulate Matter 10 at 2 months		0,179	0,227	0,125	0,166
model_ferro_2	Mother's educational level at 2 months	2	0,016	0,022	0,014	0,021
model_ferro_2	Mother's educational level at 2 months	3	0,069	0,075	0,038	0,047
model_ferro_2	Mother's educational level at 2 months	4	0,029	0,035	0,016	0,025
model_ferro_2	Mother's educational level at 2 months	5	0,014	0,020	0,000	0,008
model_ferro_2	Mother's migratory status	1	0,046	0,052	0,028	0,036
model_ferro_2	Mother's migratory status	2	0,000	0,006	0,005	0,009
model_ferro_2	Mother's migratory status	3	0,047	0,053	0,036	0,044
model_ferro_2	Mean (SD) mother's age at child's birth		0,105	0,119	0,061	0,075
model_ferro_2	Mother psychological difficulties during pregnancy		0,000	0,011	0,005	0,013
model_ferro_2	Father's educational level at 2 months	2	0,007	0,035	0,009	0,031
model_ferro_2	Father's educational level at 2 months	3	0,027	0,074	0,009	0,035
model_ferro_2	Father's educational level at 2 months	4	0,016	0,049	0,010	0,033
model_ferro_2	Father's educational level at 2 months	5	0,019	0,061	0,009	0,030
model_ferro_2	Mean (SD) number of siblings living in the house at 2 months		0,078	0,096	0,055	0,077
model_ferro_2	Mean (SD) NDVI at 2 months		0,141	0,157	0,113	0,133
model_ferro_2	Parental postnatal depression risk (EPDS scale)		0,004	0,038	0,000	0,014
model_ferro_2	Birth weight		0,010	0,015	0,004	0,007
model_ferro_2	Premature birth		0,007	0,013	0,000	0,004
model_ferro_2	Income per consumption unit at 2 months (€/month)		0,020	0,052	0,010	0,037
model_ferro_2	Rural/urban environment at 2 months		0,149	0,149	0,112	0,120
model_ferro_2	Walkability index at 2 months (tertiles)		0,113	0,124	0,055	0,082

ASMD = Absolute Standardized Mean Difference.
ASMD before and after weighting using absolute standardized mean differences for each covariate, reported as the minimum and maximum values across all imputations

Supplementary Figure 4: Histogram aircraft Model 2 (mean of weights = 0.994):

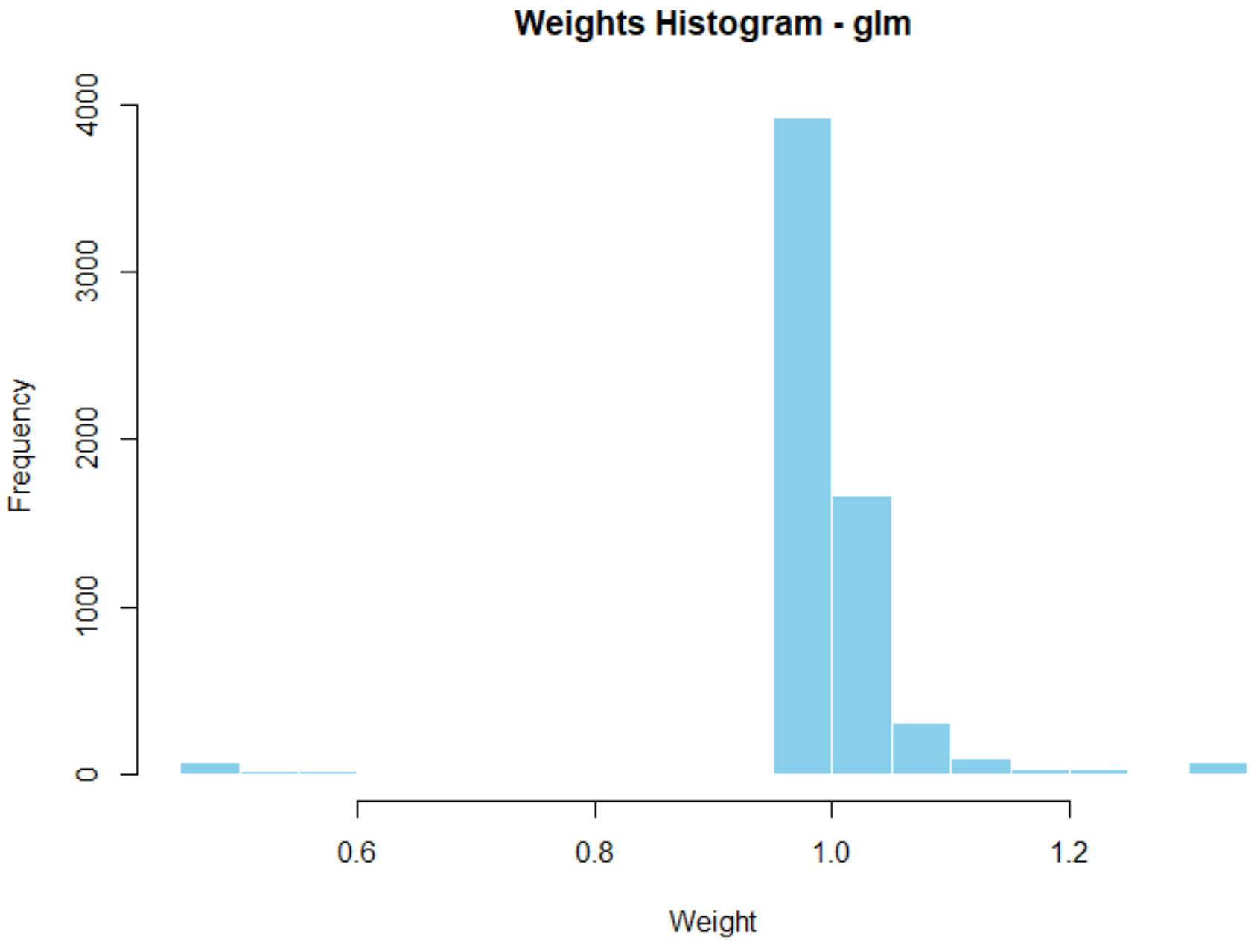

Supplementary Figure 5: Histogram railway Model 2 (mean of weights = 0.993):

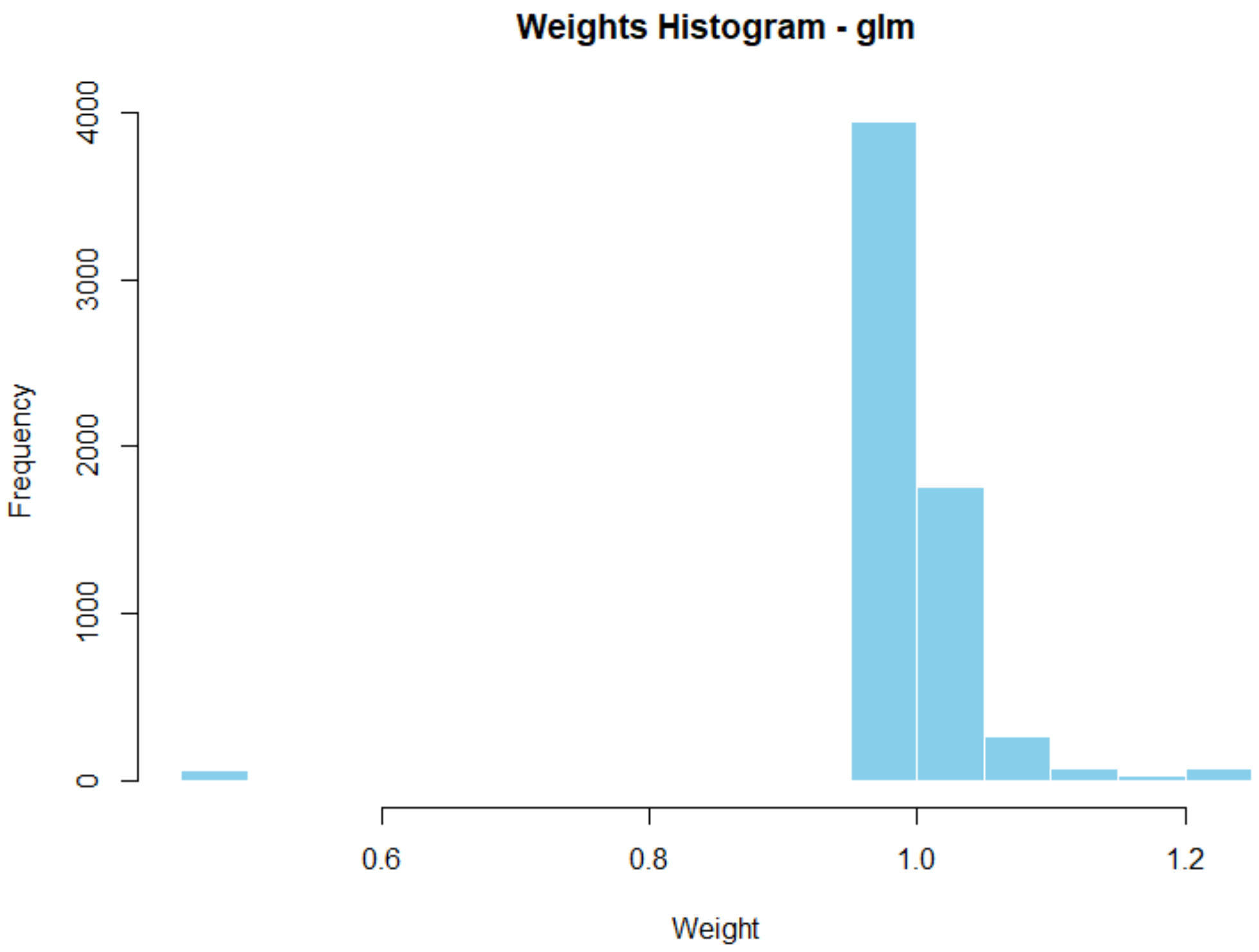

Section 6: Characteristics of the study population by exposure group at 2 months

Supplementary Table 4: Characteristics of the study population by road traffic noise at 2 months (ELFE cohort study, France, 2011-2022)

Characteristics		All participants	<55 dB(A)	>=55 dB(A)	>=60 dB(A)
Sex	Male	4044 (51.1)	1245 (50.2)	1453 (52.4)	1346 (50.6)
	Female	3870 (48.9)	1235 (49.8)	1319 (47.6)	1316 (49.4)
Birth weight	<2500 g	244 (3.1)	76 (3.1)	82 (3.0)	86 (3.2)
	≥2500 g	7511 (94.9)	2358 (95.1)	2637 (95.1)	2516 (94.5)
	Missing	159 (2.0)	46 (1.9)	53 (1.9)	60 (2.3)
Premature birth	No, <=37 weeks of amenorrhea	7098 (89.7)	2223 (89.6)	2501 (90.2)	2374 (89.2)
	Yes, >37 weeks of amenorrhea	700 (8.8)	215 (8.7)	236 (8.5)	249 (9.4)
	Missing	116 (1.5)	42 (1.7)	35 (1.3)	39 (1.5)
Mean (SD) mother's age at child's birth		31.6 (4.5)	31.7 (4.4)	31.5 (4.4)	31.6 (4.6)
Mean (SD) father's age at child's birth		33.9 (5.7)	34 (5.6)	33.9 (5.6)	34 (5.9)
Mother's educational level at 2 months	Secondary school or lower	267 (3.4)	88 (3.5)	89 (3.2)	90 (3.4)
	Secondary education (high school)	1859 (23.5)	521 (21.0)	716 (25.8)	622 (23.4)
	Higher education (Bac + 2 to +5)	1901 (24.0)	576 (23.2)	703 (25.4)	622 (23.4)
	≥5 years after high school	3839 (48.5)	1280 (51.6)	1243 (44.8)	1316 (49.4)
	Missing	48 (0.6)	15 (0.6)	21 (0.8)	12 (0.5)
Father's educational level at 2 months	Secondary school or lower	408 (5.2)	116 (4.7)	139 (5.0)	153 (5.7)
	Secondary education (high school)	2461 (31.1)	720 (29.0)	922 (33.3)	819 (30.8)
	Higher education (Bac + 2 to +5)	1469 (18.6)	437 (17.6)	571 (20.6)	461 (17.3)
	≥5 years after high school	3061 (38.7)	1038 (41.9)	966 (34.8)	1057 (39.7)
	Missing	515 (6.5)	169 (6.8)	174 (6.3)	172 (6.5)
Mother's migratory status	Not of French nationality	640 (8.1)	212 (8.5)	194 (7.0)	234 (8.8)
	French nationality descended from 1 or 2 immigrant parents	717 (9.1)	220 (8.9)	244 (8.8)	253 (9.5)
	Other French Mother	6490 (82.0)	2024 (81.6)	2308 (83.3)	2158 (81.1)
	Missing	67 (0.8)	24 (1.0)	26 (0.9)	17 (0.6)
Father's migratory status	Not of French nationality	175 (2.2)	58 (2.3)	53 (1.9)	64 (2.4)

	French nationality descended from 1 or 2 immigrant parents	1099 (13.9)	375 (15.1)	342 (12.3)	382 (14.4)
	Other French Father	5987 (75.7)	1861 (75.0)	2133 (76.9)	1993 (74.9)
	Missing	653 (8.3)	186 (7.5)	244 (8.8)	223 (8.4)
Mother psychological difficulties during pregnancy	No	6870 (86.8)	2156 (86.9)	2403 (86.7)	2311 (86.8)
	Yes	967 (12.2)	293 (11.8)	351 (12.7)	323 (12.1)
	Missing	77 (1.0)	31 (1.2)	18 (0.6)	28 (1.1)
Parental postnatal depression risk (EPDS scale)	Neither parent at risk	6359 (80.4)	2006 (80.9)	2233 (80.6)	2120 (79.6)
	One or both parent at risk	690 (8.7)	218 (8.8)	239 (8.6)	233 (8.8)
	Missing	865 (10.9)	256 (10.3)	300 (10.8)	309 (11.6)
Income per consumption unit at 2 months (€/month)		1814.3 (867.6)	1850.7 (911.7)	1775.4 (824)	1820.9 (868.3)
Parental employment at 2 months	One parent unemployed	1736 (21.9)	553 (22.3)	552 (19.9)	631 (23.7)
	Both parents unemployed	168 (2.1)	44 (1.8)	66 (2.4)	58 (2.2)
	Both parents working	5861 (74.1)	1838 (74.1)	2098 (75.7)	1925 (72.3)
	Missing	149 (1.9)	45 (1.8)	56 (2.0)	48 (1.8)
Mean (SD) number of siblings living in the house at 2 months		0.8 (0.9)	0.8 (0.9)	0.8 (0.9)	0.8 (0.9)
Railway traffic noise exposure at 2 months	<50 dB(A)	7753 (98)	2444 (98.5)	2713 (97.9)	2596 (97.5)
	>=50 dB(A)	161 (2)	36 (1.5)	59 (2.1)	66 (2.5)
Air traffic noise exposure at 2 months	<50 dB(A)	7729 (97.7)	2425 (97.8)	2709 (97.7)	2595 (97.5)
	>=50 dB(A)	185 (2.3)	55 (2.2)	63 (2.3)	67 (2.5)
Mean (SD) daily screen time at 3.5 years (hours/day)		1.1 (0.9)	1.1 (1)	1.1 (0.9)	1.1 (1)
Mean (SD) daily screen time at 5.5 years (hours/day)		1.3 (1.1)	1.3 (1.1)	1.4 (1.1)	1.3 (1.1)
Residential moving since last time-point at 3.5 years	No	6245 (78.9)	1932 (77.9)	2231 (80.5)	2082 (78.2)
	Yes	1534 (19.4)	505 (20.4)	490 (17.7)	539 (20.2)
	Missing	135 (1.7)	43 (1.7)	51 (1.8)	41 (1.5)
Residential moving since last time-point at 5.5 years	No	6226 (78.7)	1953 (78.8)	2202 (79.4)	2071 (77.8)
	Yes	1644 (20.8)	517 (20.8)	553 (19.9)	574 (21.6)
	Missing	44 (0.6)	10 (0.4)	17 (0.6)	17 (0.6)
Rural/urban environment at 2 months	< 5,000 inhabitants	2054 (26.0)	618 (24.9)	900 (32.5)	536 (20.1)
	>= 5,000 inhabitants	5793 (73.2)	1838 (74.1)	1846 (66.6)	2109 (79.2)

Missing	67 (0.8)	24 (1.0)	26 (0.9)	17 (0.6)
Mean (SD) NDVI at 2 months	0.5 (0.2)	0.5 (0.2)	0.5 (0.1)	0.5 (0.2)
Mean (SD) Particulate Matter 10 at 2 months	23.2 (4.6)	23.3 (4.6)	22.1 (4)	24.1 (4.9)
Mean (SD) SDQ external scale score at 10.5 years	4.9 (3.7)	4.9 (3.7)	4.9 (3.6)	5 (3.7)
Mean (SD) SDQ internal scale score at 10.5 years	4.1 (3.1)	4 (3)	4.1 (3)	4.2 (3.1)
Mean (SD) SDQ hyperactivity/inattention subscale score at 10.5 years	3.3 (2.6)	3.3 (2.6)	3.3 (2.6)	3.4 (2.7)
Mean (SD) SDQ conduct problems subscale score at 10.5 years	1.6 (1.6)	1.7 (1.6)	1.6 (1.5)	1.6 (1.6)
Mean (SD) SDQ emotional problems subscale score at 10.5 years	2.7 (2.2)	2.7 (2.2)	2.8 (2.2)	2.8 (2.2)
Mean (SD) SDQ peer problems subscale score at 10.5 years	1.4 (1.5)	1.3 (1.5)	1.3 (1.5)	1.5 (1.6)
SDQ hyperactivity/inattention subscale score at 10.5 years	Normal	6247 (78.9)	1969 (79.4)	2182 (78.7)
	Limit	559 (7.1)	177 (7.1)	191 (6.9)
	Abnormal	1108 (14.0)	334 (13.5)	399 (14.4)
SDQ conduct problems subscale score at 10.5 years	Normal	5956 (75.3)	1823 (73.5)	2109 (76.1)
	Limit	987 (12.5)	336 (13.5)	342 (12.3)
	Abnormal	971 (12.3)	321 (12.9)	321 (11.6)
SDQ emotional problems subscale score at 10.5 years	Normal	5316 (67.2)	1699 (68.5)	1829 (66.0)
	Limit	947 (12.0)	305 (12.3)	328 (11.8)
	Abnormal	1651 (20.9)	476 (19.2)	615 (22.2)
SDQ peer problems subscale score at 10.5 years	Normal	6440 (81.4)	2046 (82.5)	2274 (82.0)
	Limit	701 (8.9)	219 (8.8)	230 (8.3)
	Abnormal	773 (9.8)	215 (8.7)	268 (9.7)

EPDS: Edinburgh Postnatal Depression Scale
NDVI: Normalized Difference Vegetation Index
SD: Standard Deviation
SDQ: Strengths and Difficulties Questionnaire
Values represent numbers (percentages) unless stated otherwise.

Supplementary Table 5: Characteristics of the study population by railway noise at 2 months (ELFE cohort study, France, 2011-2022)

Characteristics		All participants	<50 dB(A)	>=50 dB(A)
Sex	Male	4736 (51)	4642 (50.9)	94 (52.8)
	Female	4557 (49)	4473 (49.1)	84 (47.2)
Birth weight	<2500 g	290 (3.1)	281 (3.1)	9 (5.1)
	≥2500 g	8816 (94.9)	8649 (94.9)	167 (93.8)
	Missing	187 (2.0)	185 (2.0)	2 (1.1)
Premature birth	No, <=37 weeks of amenorrhea	8334 (89.7)	8179 (89.7)	155 (87.1)
	Yes, >37 weeks of amenorrhea	826 (8.9)	804 (8.8)	22 (12.4)
	Missing	133 (1.4)	132 (1.4)	1 (0.6)
Mean (SD) mother's age at child's birth		31.5 (4.5)	31.5 (4.5)	31.4 (4.6)
Mean (SD) father's age at child's birth		33.9 (5.7)	33.9 (5.7)	34.1 (6.3)
Mother's educational level at 2 months	Secondary school or lower	351 (3.8)	347 (3.8)	4 (2.2)
	Secondary education (high school)	2292 (24.7)	2234 (24.5)	58 (32.6)
	Higher education (Bac + 2 to +5)	2239 (24.1)	2205 (24.2)	34 (19.1)
	≥5 years after high school	4345 (46.8)	4265 (46.8)	80 (44.9)
	Missing	66 (0.7)	64 (0.7)	2 (1.1)
Father's educational level at 2 months	Secondary school or lower	489 (5.3)	476 (5.2)	13 (7.3)
	Secondary education (high school)	3017 (32.5)	2952 (32.4)	65 (36.5)
	Higher education (Bac + 2 to +5)	1715 (18.5)	1689 (18.5)	26 (14.6)
	≥5 years after high school	3409 (36.7)	3357 (36.8)	52 (29.2)
	Missing	663 (7.1)	641 (7.0)	22 (12.4)
Mother's migratory status	Not of French nationality	726 (7.8)	705 (7.7)	21 (11.8)
	French nationality descended from 1 or 2 immigrant parents	837 (9.0)	820 (9.0)	17 (9.6)
	Other French Mother	7640 (82.2)	7503 (82.3)	137 (77.0)
	Missing	90 (1.0)	87 (1.0)	3 (1.7)
Father's migratory status	Not of French nationality	220 (2.4)	210 (2.3)	10 (5.6)
	French nationality descended from 1 or 2 immigrant parents	1239 (13.3)	1204 (13.2)	35 (19.7)

	Other French Father	7009 (75.4)	6897 (75.7)	112 (62.9)
	Missing	825 (8.9)	804 (8.8)	21 (11.8)
Mother psychological difficulties during pregnancy	No	8097 (87.1)	7943 (87.1)	154 (86.5)
	Yes	1111 (12.0)	1089 (11.9)	22 (12.4)
	Missing	85 (0.9)	83 (0.9)	2 (1.1)
Parental postnatal depression risk (EPDS scale)	Neither parent at risk	7380 (79.4)	7251 (79.6)	129 (72.5)
	One or both parent at risk	810 (8.7)	792 (8.7)	18 (10.1)
	Missing	1103 (11.9)	1072 (11.8)	31 (17.4)
Income per consumption unit at 2 months (€/month)		1779.3 (868.2)	1780.4 (871.8)	1720.6 (656.8)
Parental employment at 2 months	One parent unemployed	2084 (22.4)	2046 (22.4)	38 (21.3)
	Both parents unemployed	211 (2.3)	207 (2.3)	4 (2.2)
	Both parents working	6806 (73.2)	6675 (73.2)	131 (73.6)
	Missing	192 (2.1)	187 (2.1)	5 (2.8)
Mean (SD) number of siblings living in the house at 2 months		0.8 (0.9)	0.8 (0.9)	0.7 (0.8)
Road traffic noise exposure at 2 months	<55 dB(A)	2923 (31.5)	2884 (31.6)	39 (21.9)
	>=55 dB(A)	3294 (35.4)	3228 (35.4)	66 (37.1)
	>=60 dB(A)	3076 (33.1)	3003 (32.9)	73 (41.0)
Air traffic noise exposure at 2 months	<50 dB(A)	9097 (97.9)	8923 (97.9)	174 (97.8)
	>=50 dB(A)	196 (2.1)	192 (2.1)	4 (2.2)
Mean (SD) daily screen time at 3.5 years (hours/day)		1.1 (0.9)	1.1 (0.9)	1.2 (0.9)
Mean (SD) daily screen time at 5.5 years (hours/day)		1.3 (1.1)	1.3 (1.1)	1.6 (1.3)
Residential moving since last time-point at 3.5 years	No	7145 (76.9)	7015 (77.0)	130 (73.0)
	Yes	1683 (18.1)	1642 (18.0)	41 (23.0)
	Missing	465 (5.0)	458 (5.0)	7 (3.9)
Residential moving since last time-point at 5.5 years	No	7050 (75.9)	6927 (76.0)	123 (69.1)
	Yes	1900 (20.4)	1852 (20.3)	48 (27.0)
	Missing	343 (3.7)	336 (3.7)	7 (3.9)
Rural/urban environment at 2 months	< 5,000 inhabitants	2705 (29.1)	2687 (29.5)	18 (10.1)
	>= 5,000 inhabitants	6498 (69.9)	6341 (69.6)	157 (88.2)

	Missing	90 (1.0)	87 (1.0)	3 (1.7)
Mean (SD) NDVI at 2 months		0.5 (0.2)	0.5 (0.2)	0.5 (0.1)
Mean (SD) Particulate Matter 10 at 2 months		22.9 (4.6)	22.9 (4.7)	23.7 (3.3)
Mean (SD) SDQ external scale score at 10.5 years		4.9 (3.7)	4.9 (3.7)	4.8 (3.4)
Mean (SD) SDQ internal scale score at 10.5 years		4.1 (3.1)	4.1 (3.1)	3.5 (2.7)
Mean (SD) SDQ hyperactivity/inattention subscale score at 10.5 years		3.3 (2.6)	3.3 (2.6)	3.3 (2.6)
Mean (SD) SDQ conduct problems subscale score at 10.5 years		1.6 (1.6)	1.6 (1.6)	1.5 (1.4)
Mean (SD) SDQ emotional problems subscale score at 10.5 years		2.7 (2.2)	2.8 (2.2)	2.3 (2)
Mean (SD) SDQ peer problems subscale score at 10.5 years		1.4 (1.5)	1.4 (1.5)	1.1 (1.3)
SDQ hyperactivity/inattention subscale score at 10.5 years	Normal	6247 (78.9)	6119 (78.9)	128 (79.5)
	Limit	559 (7.1)	545 (7.0)	14 (8.7)
	Abnormal	1108 (14.0)	1089 (14.0)	19 (11.8)
SDQ conduct problems subscale score at 10.5 years	Normal	5956 (75.3)	5826 (75.1)	130 (80.7)
	Limit	987 (12.5)	970 (12.5)	17 (10.6)
	Abnormal	971 (12.3)	957 (12.3)	14 (8.7)
SDQ emotional problems subscale score at 10.5 years	Normal	5316 (67.2)	5201 (67.1)	115 (71.4)
	Limit	947 (12.0)	926 (11.9)	21 (13.0)
	Abnormal	1651 (20.9)	1626 (21.0)	25 (15.5)
SDQ peer problems subscale score at 10.5 years	Normal	6440 (81.4)	6306 (81.3)	134 (83.2)
	Limit	701 (8.9)	682 (8.8)	19 (11.8)
	Abnormal	773 (9.8)	765 (9.9)	8 (5.0)

EPDS: Edinburgh Postnatal Depression Scale
NDVI: Normalized Difference Vegetation Index
SD: Standard Deviation
SDQ: Strengths and Difficulties Questionnaire
Values represent numbers (percentages) unless stated otherwise.

Supplementary Table 6: Characteristics of the study population by aircraft noise at 2 months (ELFE cohort study, France, 2011-2022)

Characteristics		All participants	<50 dB(A)	>=50 dB(A)
Sex	Male	4736 (51)	4638 (51)	98 (50)
	Female	4557 (49)	4459 (49)	98 (50)
Birth weight	<2500 g	290 (3.1)	286 (3.1)	4 (2.0)
	≥2500 g	8816 (94.9)	8626 (94.8)	190 (96.9)
	Missing	187 (2.0)	185 (2.0)	2 (1.0)
Premature birth	No, <=37 weeks of amenorrhea	8334 (89.7)	8155 (89.6)	179 (91.3)
	Yes, >37 weeks of amenorrhea	826 (8.9)	810 (8.9)	16 (8.2)
	Missing	133 (1.4)	132 (1.5)	1 (0.5)
Mean (SD) mother's age at child's birth		31.5 (4.5)	31.5 (4.5)	31.3 (4.8)
Mean (SD) father's age at child's birth		33.9 (5.7)	33.9 (5.7)	33.6 (5.7)
Mother's educational level at 2 months	Secondary school or lower	351 (3.8)	333 (3.7)	18 (9.2)
	Secondary education (high school)	2292 (24.7)	2255 (24.8)	37 (18.9)
	Higher education (Bac + 2 to +5)	2239 (24.1)	2199 (24.2)	40 (20.4)
	≥5 years after high school	4345 (46.8)	4248 (46.7)	97 (49.5)
	Missing	66 (0.7)	62 (0.7)	4 (2.0)
Father's educational level at 2 months	Secondary school or lower	489 (5.3)	471 (5.2)	18 (9.2)
	Secondary education (high school)	3017 (32.5)	2963 (32.6)	54 (27.6)
	Higher education (Bac + 2 to +5)	1715 (18.5)	1688 (18.6)	27 (13.8)
	≥5 years after high school	3409 (36.7)	3336 (36.7)	73 (37.2)
	Missing	663 (7.1)	639 (7.0)	24 (12.2)
Mother's migratory status	Not of French nationality	726 (7.8)	696 (7.7)	30 (15.3)
	French nationality descended from 1 or 2 immigrant parents	837 (9.0)	803 (8.8)	34 (17.3)
	Other French Mother	7640 (82.2)	7513 (82.6)	127 (64.8)
	Missing	90 (1.0)	85 (0.9)	5 (2.6)
Father's migratory status	Not of French nationality	220 (2.4)	209 (2.3)	11 (5.6)
	French nationality descended from 1 or 2 immigrant parents	1239 (13.3)	1200 (13.2)	39 (19.9)

	Other French Father	7009 (75.4)	6888 (75.7)	121 (61.7)
	Missing	825 (8.9)	800 (8.8)	25 (12.8)
Mother psychological difficulties during pregnancy	No	8097 (87.1)	7925 (87.1)	172 (87.8)
	Yes	1111 (12.0)	1089 (12.0)	22 (11.2)
	Missing	85 (0.9)	83 (0.9)	2 (1.0)
Parental postnatal depression risk (EPDS scale)	Neither parent at risk	7380 (79.4)	7234 (79.5)	146 (74.5)
	One or both parent at risk	810 (8.7)	798 (8.8)	12 (6.1)
	Missing	1103 (11.9)	1065 (11.7)	38 (19.4)
Income per consumption unit at 2 months (€/month)		1779.3 (868.2)	1777.5 (867.8)	1860.1 (888.3)
Parental employment at 2 months	One parent unemployed	2084 (22.4)	2046 (22.5)	38 (19.4)
	Both parents unemployed	211 (2.3)	207 (2.3)	4 (2.0)
	Both parents working	6806 (73.2)	6660 (73.2)	146 (74.5)
	Missing	192 (2.1)	184 (2.0)	8 (4.1)
Mean (SD) number of siblings living in the house at 2 months		0.8 (0.9)	0.8 (0.9)	0.8 (0.9)
Road traffic noise exposure at 2 months	<55 dB(A)	2923 (31.5)	2866 (31.5)	57 (29.1)
	>=55 dB(A)	3294 (35.4)	3226 (35.5)	68 (34.7)
	>=60 dB(A)	3076 (33.1)	3005 (33.0)	71 (36.2)
Railway traffic noise exposure at 2 months	<50 dB(A)	9115 (98.1)	8923 (98.1)	192 (98.0)
	>=50 dB(A)	178 (1.9)	174 (1.9)	4 (2.0)
Mean (SD) daily screen time at 3.5 years (hours/day)		1.1 (0.9)	1.1 (0.9)	1.1 (1.1)
Mean (SD) daily screen time at 5.5 years (hours/day)		1.3 (1.1)	1.3 (1.1)	1.4 (1.3)
Residential moving since last time-point at 3.5 years	No	7145 (76.9)	7003 (77.0)	142 (72.4)
	Yes	1683 (18.1)	1639 (18.0)	44 (22.4)
	Missing	465 (5.0)	455 (5.0)	10 (5.1)
Residential moving since last time-point at 5.5 years	No	7050 (75.9)	6894 (75.8)	156 (79.6)
	Yes	1900 (20.4)	1865 (20.5)	35 (17.9)
	Missing	343 (3.7)	338 (3.7)	5 (2.6)
Rural/urban environment at 2 months	< 5,000 inhabitants	2705 (29.1)	2693 (29.6)	12 (6.1)
	>= 5,000 inhabitants	6498 (69.9)	6319 (69.5)	179 (91.3)

	Missing	90 (1.0)	85 (0.9)	5 (2.6)
Mean (SD) NDVI at 2 months		0.5 (0.2)	0.5 (0.2)	0.5 (0.1)
Mean (SD) Particulate Matter 10 at 2 months		22.9 (4.6)	22.9 (4.6)	24.3 (4.2)
Mean (SD) SDQ external scale score at 10.5 years		4.9 (3.7)	4.9 (3.7)	4.9 (3.6)
Mean (SD) SDQ internal scale score at 10.5 years		4.1 (3.1)	4.1 (3.1)	4.7 (3.2)
Mean (SD) SDQ hyperactivity/inattention subscale score at 10.5 years		3.3 (2.6)	3.3 (2.6)	3.3 (2.6)
Mean (SD) SDQ conduct problems subscale score at 10.5 years		1.6 (1.6)	1.6 (1.6)	1.7 (1.6)
Mean (SD) SDQ emotional problems subscale score at 10.5 years		2.7 (2.2)	2.7 (2.2)	3 (2.2)
Mean (SD) SDQ peer problems subscale score at 10.5 years		1.4 (1.5)	1.4 (1.5)	1.8 (1.7)
SDQ hyperactivity/inattention subscale score at 10.5 years	Normal	6247 (78.9)	6098 (78.9)	149 (80.5)
	Limit	559 (7.1)	547 (7.1)	12 (6.5)
	Abnormal	1108 (14.0)	1084 (14.0)	24 (13.0)
SDQ conduct problems subscale score at 10.5 years	Normal	5956 (75.3)	5825 (75.4)	131 (70.8)
	Limit	987 (12.5)	961 (12.4)	26 (14.1)
	Abnormal	971 (12.3)	943 (12.2)	28 (15.1)
SDQ emotional problems subscale score at 10.5 years	Normal	5316 (67.2)	5204 (67.3)	112 (60.5)
	Limit	947 (12.0)	915 (11.8)	32 (17.3)
	Abnormal	1651 (20.9)	1610 (20.8)	41 (22.2)
SDQ peer problems subscale score at 10.5 years	Normal	6440 (81.4)	6305 (81.6)	135 (73.0)
	Limit	701 (8.9)	682 (8.8)	19 (10.3)
	Abnormal	773 (9.8)	742 (9.6)	31 (16.8)

EPDS: Edinburgh Postnatal Depression Scale
NDVI: Normalized Difference Vegetation Index
SD: Standard Deviation
SDQ: Strengths and Difficulties Questionnaire
Values represent numbers (percentages) unless stated otherwise.

Section 7: Description of SDQ scores across exposure categories.

Supplementary Table 7: Mean (SD) SDQ scores across traffic noise exposure groups (ELFE cohort study, France, 2011–2022)

SDQ scale	Overall sample (N=7914)	Persistent low road <55 dB(A) (N=1540)	Persistent high road >=60 dB(A) (N=1630)	Persistent low rail <50 dB(A) (N=7700)	High rail at any timepoint >=50 dB(A) (N=214)	Persistent low aircraft <50 dB(A) (N=7645)	High aircraft at any timepoint >=50 dB(A) (N=269)
SDQ external scale	4.93 (3.68)	4.82 (3.58)	5.03 (3.78)	4.93 (3.68)	4.82 (3.59)	4.93 (3.68)	4.97 (3.52)
SDQ internal scale	4.13 (3.05)	3.90 (2.92)	4.19 (3.14)	4.14 (3.06)	3.73 (2.86)	4.11 (3.05)	4.50 (3.13)
SDQ hyperactivity	3.32 (2.65)	3.22 (2.59)	3.41 (2.70)	3.32 (2.65)	3.34 (2.69)	3.32 (2.66)	3.29 (2.48)
SDQ conduct problems	1.61 (1.57)	1.60 (1.54)	1.62 (1.62)	1.61 (1.57)	1.48 (1.42)	1.61 (1.57)	1.68 (1.60)
SDQ emotional symptoms	2.75 (2.20)	2.62 (2.15)	2.75 (2.21)	2.76 (2.20)	2.49 (2.05)	2.74 (2.20)	2.90 (2.18)
SDQ peer relationship problems	1.38 (1.52)	1.29 (1.45)	1.44 (1.59)	1.38 (1.52)	1.24 (1.40)	1.37 (1.51)	1.60 (1.69)

SD: Standard Deviation

SDQ: Strengths and Difficulties Questionnaire

Supplementary Table 8: Distribution of SDQ categories according to traffic noise exposure groups at age 10.5 years (ELFE cohort study, France, 2011-2022)

SDQ scale	SDQ category	Overall sample (N=7914)	Persistent low road <55 dB(A) (N=1540)	Persistent high road >=60 dB(A) (N=1630)	Persistent low rail <50 dB(A) (N=7700)	High rail at any timepoint >=50 dB(A) (N=214)	Persistent low aircraft <50 dB(A) (N=7645)	High aircraft at any timepoint >=50 dB(A) (N=269)
SDQ hyperactivity	Normal	6247 (78.9%)	1235 (80.2%)	1274 (78.2%)	6081 (79.0%)	166 (77.6%)	6028 (78.8%)	219 (81.4%)
	Limit	559 (7.1%)	109 (7.1%)	119 (7.3%)	540 (7.0%)	19 (8.9%)	542 (7.1%)	17 (6.3%)
	Abnormal	1108 (14.0%)	196 (12.7%)	237 (14.5%)	1079 (14.0%)	29 (13.6%)	1075 (14.1%)	33 (12.3%)
SDQ conduct problems	Normal	5956 (75.3%)	1149 (74.6%)	1227 (75.3%)	5787 (75.2%)	169 (79.0%)	5764 (75.4%)	192 (71.4%)
	Limit	987 (12.5%)	210 (13.6%)	199 (12.2%)	964 (12.5%)	23 (10.7%)	950 (12.4%)	37 (13.8%)
	Abnormal	971 (12.3%)	181 (11.8%)	204 (12.5%)	949 (12.3%)	22 (10.3%)	931 (12.2%)	40 (14.9%)
SDQ emotional symptoms	Normal	5316 (67.2%)	1090 (70.8%)	1103 (67.7%)	5168 (67.1%)	148 (69.2%)	5145 (67.3%)	171 (63.6%)
	Limit	947 (12.0%)	179 (11.6%)	190 (11.7%)	920 (11.9%)	27 (12.6%)	907 (11.9%)	40 (14.9%)
	Abnormal	1651 (20.9%)	271 (17.6%)	337 (20.7%)	1612 (20.9%)	39 (18.2%)	1593 (20.8%)	58 (21.6%)
SDQ peer relationship problems	Normal	6440 (81.4%)	1288 (83.6%)	1307 (80.2%)	6265 (81.4%)	175 (81.8%)	6239 (81.6%)	201 (74.7%)
	Limit	701 (8.9%)	125 (8.1%)	148 (9.1%)	678 (8.8%)	23 (10.7%)	673 (8.8%)	28 (10.4%)
	Abnormal	773 (9.8%)	127 (8.2%)	175 (10.7%)	757 (9.8%)	16 (7.5%)	733 (9.6%)	40 (14.9%)

SD: Standard Deviation
SDQ: Strengths and Difficulties Questionnaire
Values represent numbers (percentages).

Section 8: Stratification by rural/urban area

Supplementary Table 9: Marginal structural model estimates of the effect of persistent high (≥60 dB(A)) versus low (<55 dB(A)) road traffic noise exposure across childhood on children's emotional and behavioral difficulties at 10·5 years – stratification by rural/urban area (ELFE cohort, 2011–2022)

	Rural		Urban	
**SDQ scale**	**β (95% CI)**	**P value**	**β (95% CI)**	**P value**
SDQ external scale	0.73 (0.09, 1.36)	0.02	-0.04 (-0.38, 0.30)	0.82
SDQ internal scale	0.84 (0.31, 1.36)	<0.01	0.30 (-0.00, 0.60)	0.051
SDQ hyperactivity	0.37 (-0.07, 0.81)	0.10	0.06 (-0.18, 0.30)	0.64
SDQ conduct problems	0.36 (0.08, 0.64)	0.01	-0.10 (-0.25, 0.06)	0.22
SDQ emotional symptoms	0.49 (0.13, 0.85)	0.01	0.19 (-0.03, 0.40)	0.08
SDQ peer relationship problems	0.35 (0.05, 0.64)	0.02	0.11 (-0.04, 0.26)	0.16

Estimates represent counterfactual contrasts between exposure histories in the weighted population and should not be interpreted as simple comparisons between observed exposure groups.

SDQ = Strengths and Difficulties Questionnaire, β = Adjusted regression coefficient, CI = Confidence Interval.

Section 9: Analyses considering thresholds of SDQ

According to existing guidelines (18), the SDQ normal, borderline, and abnormal score ranges are as follows:

- Hyperactivity/inattention: normal (0-5), borderline (6), abnormal (7-10).

- Conduct problems: normal (0-2), borderline (3), abnormal (4-10).

- Emotional symptoms: normal (0-3), borderline (4), abnormal (5-10).

- Peer relationship problems: normal (0-2), borderline (3), abnormal (4-10).

To allow classification into validated diagnostic categories, logistic regressions considering SDQ thresholds for each subscale and comparing with a normal score were performed. We modelled two separate binary outcomes (borderline/abnormal *vs* normal, and abnormal *vs* normal/borderline).

Supplementary Table 10: Marginal structural model estimates of the effect of persistent high (≥60 dB(A)) versus low (<55 dB(A)) road traffic noise exposure across childhood on children's emotional and behavioral difficulties at 10·5 years - considering thresholds of SDQ (ELFE cohort, 2011–2022)

	**Model 1**		**Model 2**	
**SDQ subscale**	**OR (95% CI)**	**P value**	**OR (95% CI)**	**P value**
SDQ hyperactivity limit	1.03 (0.99, 1.06)	0.10	1.02 (0.99, 1.06)	0.15
SDQ hyperactivity abnormal	1.01 (0.99, 1.04)	0.39	1.01 (0.98, 1.04)	0.41
SDQ conduct problems limit	0.99 (0.96, 1.02)	0.48	0.99 (0.95, 1.02)	0.43
SDQ conduct problems abnormal	1.01 (0.98, 1.03)	0.67	1.01 (0.98, 1.04)	0.48
SDQ emotional symptoms limit	1.03 (0.99, 1.07)	0.09	1.03 (1.00, 1.07)	0.08
SDQ emotional symptoms abnormal	1.02 (0.99, 1.05)	0.16	1.02 (0.99, 1.06)	0.17
SDQ peer relationship problems limit	1.03 (1.00, 1.07)	0.03	1.04 (1.01, 1.08)	0.02
SDQ peer relationship problems abnormal	1.04 (1.01, 1.06)	<0.01	1.04 (1.01, 1.07)	<0.01

Estimates represent counterfactual contrasts between exposure histories in the weighted population and should not be interpreted as simple comparisons between observed exposure groups.

SDQ = Strengths and Difficulties Questionnaire, OR = Odds Ratio, CI = Confidence Interval.

Supplementary Table 11: Marginal structural model estimates of the effect of childhood exposure to aircraft and railway noise (≥50 dB(A)) at any timepoint and children's emotional and behavioral difficulties at 10·5 years - considering thresholds of SDQ (ELFE cohort, 2011-2022)

	Model 1		Model 2	
**SDQ scale**	**OR (95% CI)**	**P value**	**OR (95% CI)**	**P value**
**Aircraft noise**				
SDQ hyperactivity limit	0.74 (0.50, 1.10)	0.13	0.72 (0.49, 1.06)	0.10
SDQ hyperactivity abnormal	0.74 (0.47, 1.19)	0.21	0.73 (0.46, 1.15)	0.17
SDQ conduct problems limit	1.29 (0.92, 1.82)	0.14	1.21 (0.87, 1.71)	0.26
SDQ conduct problems abnormal	1.43 (0.93, 2.22)	0.11	1.35 (0.88, 2.08)	0.17
SDQ emotional symptoms limit	1.39 (1.00, 1.92)	0.05	1.33 (0.96, 1.85)	0.09
SDQ emotional symptoms abnormal	1.05 (0.71, 1.55)	0.80	1.03 (0.70, 1.51)	0.89
SDQ peer relationship problems limit	1.41 (0.97, 2.04)	0.07	1.39 (0.96, 2.01)	0.08
SDQ peer relationship problems abnormal	1.77 (1.13, 2.76)	0.01	1.70 (1.09, 2.65)	0.02
**Railway noise**				
SDQ hyperactivity limit	0.92 (0.61, 1.40)	0.70	0.93 (0.60, 1.44)	0.74
SDQ hyperactivity abnormal	0.84 (0.50, 1.42)	0.52	0.89 (0.51, 1.57)	0.70
SDQ conduct problems limit	0.76 (0.50, 1.18)	0.22	0.81 (0.51, 1.28)	0.37
SDQ conduct problems abnormal	0.80 (0.47, 1.39)	0.43	0.85 (0.49, 1.46)	0.55
SDQ emotional symptoms limit	0.83 (0.57, 1.20)	0.32	0.81 (0.55, 1.19)	0.29
SDQ emotional symptoms abnormal	0.62 (0.41, 0.95)	0.03	0.60 (0.39, 0.92)	0.02
SDQ peer relationship problems limit	0.91 (0.57, 1.46)	0.70	0.98 (0.59, 1.63)	0.94
SDQ peer relationship problems abnormal	0.65 (0.34, 1.27)	0.21	0.69 (0.34, 1.41)	0.31

Estimates represent counterfactual contrasts between exposure histories in the weighted population and should not be interpreted as simple comparisons between observed exposure groups.

SDQ = Strengths and Difficulties Questionnaire, OR = Odds Ratio, CI = Confidence Interval.

Section 10: Complete case analyses

Supplementary Table 12: Marginal structural model estimates of the effect of persistent high (≥60 dB(A)) versus low (<55 dB(A)) road traffic noise exposure across childhood on children's emotional and behavioral difficulties at 10·5 years – complete case (ELFE cohort, 2011–2022)

	**Model 1**		**Model 2**	
**SDQ scale**	**β (95% CI)**	**P value**	**β (95% CI)**	**P value**
SDQ externalising symptoms	0.16 (-0.14, 0.47)	0.29	0.20 (-0.13, 0.53)	0.23
SDQ internalising symptoms	0.58 (0.30, 0.86)	<0.01	0.59 (0.30, 0.87)	<0.01
SDQ hyperactivity	0.15 (-0.07, 0.37)	0.17	0.18 (-0.05, 0.42)	0.13
SDQ conduct problems	0.01 (-0.12, 0.15)	0.86	0.02 (-0.12, 0.16)	0.80
SDQ emotional symptoms	0.31 (0.12, 0.50)	<0.01	0.29 (0.09, 0.49)	<0.01
SDQ peer relationship problems	0.27 (0.13, 0.41)	<0.01	0.30 (0.15, 0.45)	<0.01

Estimates represent counterfactual contrasts between exposure histories in the weighted population and should not be interpreted as simple comparisons between observed exposure groups.

SDQ = Strengths and Difficulties Questionnaire, β = Adjusted regression coefficient, CI = Confidence Interval.

Supplementary Table 13: Marginal structural model estimates of the effect of childhood exposure to aircraft and railway noise (≥50 dB(A)) at any timepoint and children's emotional and behavioral difficulties at 10·5 years – complete case (ELFE cohort, 2011-2022)

	**Model 1**		**Model 2**	
**SDQ scale**	**β (95% CI)**	**P value**	**β (95% CI)**	**P value**
Aircraft noise				
SDQ externalising symptoms	0.02 (-0.65, 0.69)	0.95	-0.06 (-0.69, 0.57)	0.85
SDQ internalising symptoms	0.46 (-0.10, 1.02)	0.11	0.39 (-0.17, 0.94)	0.17
SDQ hyperactivity	-0.14 (-0.54, 0.26)	0.49	-0.17 (-0.57, 0.24)	0.42
SDQ conduct problems	0.16 (-0.22, 0.55)	0.41	0.10 (-0.24, 0.45)	0.55
SDQ emotional symptoms	0.20 (-0.15, 0.55)	0.27	0.15 (-0.21, 0.50)	0.42
SDQ peer relationship problems	0.27 (-0.10, 0.63)	0.15	0.24 (-0.11, 0.59)	0.18
Railway noise				
SDQ externalising symptoms	-0.77 (-1.38, -0.17)	0.01	-0.78 (-1.41, -0.15)	0.01
SDQ internalising symptoms	-0.55 (-1.10, -0.01)	0.05	-0.58 (-1.10, -0.05)	0.03
SDQ hyperactivity	-0.45 (-0.90, -0.00)	0.05	-0.45 (-0.91, 0.01)	0.06
SDQ conduct problems	-0.33 (-0.58, -0.07)	0.01	-0.33 (-0.59, -0.07)	0.01
SDQ emotional symptoms	-0.32 (-0.69, 0.05)	0.09	-0.34 (-0.71, 0.03)	0.07
SDQ peer relationship problems	-0.23 (-0.51, 0.05)	0.11	-0.23 (-0.50, 0.04)	0.09

Estimates represent counterfactual contrasts between exposure histories in the weighted population and should not be interpreted as simple comparisons between observed exposure groups.

SDQ = Strengths and Difficulties Questionnaire, β = Adjusted regression coefficient, CI = Confidence Interval.

Section 11: Stepwise regression models

We fitted a sequence of regression models with stepwise adjustment for baseline covariates and examined attenuation of the exposure coefficient across models compared to crude model. Exposure was defined at baseline only. The model sequence was defined as follows:

1. Crude model.
2. + child baseline covariates: sex, child age when SDQ was reported, weight at birth.
3. + parental covariates: parent's education level, family income per unit of consumption, parental employment, parental age at child's birth, parent's migratory status, parental postnatal depression.
4. + perinatal factors: prematurity, mother psychological difficulties during pregnancy.

As shown below, percentage attenuation of the exposure coefficient was generally modest (typically <25%), suggesting limited confounding by time-invariant factors.

<u>Supplementary Table 14: Associations between exposure to residential noise and children's emotional and behavioral difficulties at 10·5 years – stepwise regression models (ELFE cohort, 2011-2022)</u>

		Model 1 (crude)		Model 2 (+ child baseline)			Model 3 (+ parental baseline)			Model 4 (+ perinatal factors)		
**SDQ scale**	**Exposure**	**β**	**P value**	**β**	**P value**	**% att.**	**β**	**P value**	**% att.**	**β**	**P value**	**% att.**
SDQ externalising symptoms	Road noise >=60 dB(A)	0.17 (-0.06, 0.39)	0.15	0.13 (-0.09, 0.35)	0.24	23.53	0.14 (-0.08, 0.36)	0.20	17.65	0.14 (-0.08, 0.35)	0.22	17.65
SDQ internalising symptoms	Road noise >=60 dB(A)	0.27 (0.08, 0.47)	0.01	0.27 (0.08, 0.47)	0.01	0	0.28 (0.09, 0.47)	<0.01	-3.7	0.28 (0.09, 0.47)	<0.01	-3.7
SDQ hyperactivity	Road noise >=60 dB(A)	0.12 (-0.04, 0.28)	0.15	0.09 (-0.06, 0.25)	0.25	25	0.10 (-0.05, 0.26)	0.19	16.67	0.10 (-0.05, 0.25)	0.20	16.67
SDQ conduct problems	Road noise >=60 dB(A)	0.05 (-0.05, 0.15)	0.37	0.04 (-0.06, 0.14)	0.43	20	0.04 (-0.06, 0.14)	0.45	20	0.04 (-0.06, 0.13)	0.47	20
SDQ emotional symptoms	Road noise >=60 dB(A)	0.07 (-0.07, 0.21)	0.31	0.07 (-0.07, 0.21)	0.31	0	0.08 (-0.05, 0.22)	0.22	-14.29	0.08 (-0.05, 0.21)	0.24	-14.29
SDQ peer relationship problems	Road noise >=60 dB(A)	0.20 (0.10, 0.31)	<0.01	0.20 (0.10, 0.31)	<0.01	0	0.20 (0.10, 0.30)	<0.01	0	0.20 (0.10, 0.30)	<0.01	0
SDQ externalising symptoms	Rail noise >=50 dB(A)	-0.64 (-1.38, 0.09)	0.09	-0.64 (-1.37, 0.09)	0.09	0	-0.70 (-1.42, 0.02)	0.06	-9.37	-0.66 (-1.37, 0.05)	0.07	-3.13
SDQ internalising symptoms	Rail noise >=50 dB(A)	-0.96 (-1.48, -0.45)	<0.01	-0.97 (-1.48, -0.45)	<0.01	-1.04	-0.97 (-1.52, -0.42)	<0.01	-1.04	-0.94 (-1.49, -0.39)	<0.01	2.08
SDQ hyperactivity	Rail noise >=50 dB(A)	-0.38 (-0.89, 0.12)	0.14	-0.37 (-0.87, 0.12)	0.14	2.63	-0.41 (-0.90, 0.07)	0.09	-7.89	-0.39 (-0.87, 0.09)	0.11	-2.63
SDQ conduct problems	Rail noise >=50 dB(A)	-0.26 (-0.58, 0.05)	0.10	-0.26 (-0.58, 0.05)	0.10	0	-0.29 (-0.61, 0.02)	0.07	-11.54	-0.27 (-0.58, 0.04)	0.09	-3.85
SDQ emotional symptoms	Rail noise >=50 dB(A)	-0.65 (-1.01, -0.29)	<0.01	-0.64 (-1.00, -0.28)	<0.01	1.54	-0.62 (-0.98, -0.27)	<0.01	4.62	-0.60 (-0.96, -0.25)	<0.01	7.69
SDQ peer relationship problems	Rail noise >=50 dB(A)	-0.32 (-0.58, -0.05)	0.02	-0.32 (-0.59, -0.06)	0.02	0	-0.35 (-0.63, -0.06)	0.02	-9.37	-0.34 (-0.62, -0.06)	0.02	-6.25
SDQ externalising symptoms	Aero noise >=50 dB(A)	0.14 (-0.57, 0.84)	0.70	0.09 (-0.58, 0.76)	0.79	35.71	0.17 (-0.50, 0.85)	0.61	-21.43	0.19 (-0.48, 0.86)	0.58	-35.71
SDQ internalising symptoms	Aero noise >=50 dB(A)	0.84 (0.16, 1.52)	0.02	0.85 (0.17, 1.52)	0.01	-1.19	0.85 (0.22, 1.47)	0.01	-1.19	0.86 (0.22, 1.49)	0.01	-2.38
SDQ hyperactivity	Aero noise >=50 dB(A)	-0.09 (-0.56, 0.38)	0.72	-0.12 (-0.56, 0.32)	0.58	-33.33	-0.05 (-0.49, 0.40)	0.84	44.44	-0.04 (-0.48, 0.40)	0.87	55.56
SDQ conduct problems	Aero noise >=50 dB(A)	0.22 (-0.14, 0.59)	0.23	0.22 (-0.15, 0.58)	0.25	0	0.22 (-0.14, 0.58)	0.23	0	0.23 (-0.13, 0.58)	0.22	-4.55
SDQ emotional symptoms	Aero noise >=50 dB(A)	0.26 (-0.19, 0.72)	0.26	0.27 (-0.18, 0.72)	0.24	-3.85	0.35 (-0.08, 0.78)	0.11	-34.62	0.36 (-0.08, 0.79)	0.11	-38.46
SDQ peer relationship problems	Aero noise >=50 dB(A)	0.58 (0.20, 0.95)	<0.01	0.58 (0.20, 0.95)	<0.01	0	0.50 (0.15, 0.84)	<0.01	13.79	0.50 (0.15, 0.85)	<0.01	13.79

SDQ = Strengths and Difficulties Questionnaire, β = Adjusted regression coefficient, CI = Confidence Interval.

Section 12: Additionally-adjusted models for externalizing and internalizing difficulties at 5.5 years

As a sensitivity analysis, we additionally adjusted for externalizing and internalizing difficulties at age 5.5 years to partially account for early-life vulnerability and familial factors. While such adjustment may introduce bias, as earlier mental health may lie on the causal pathway between exposure and later outcomes, results were not attenuated. In contrast, the association between aircraft noise exposure and internalizing symptoms was strengthened and reached statistical significance after adjustment. This pattern may reflect a reduction of non-specific variability or residual confounding, however, because earlier mental health lies on the causal pathway, this adjustment could also induce over-adjustment or collider bias. These results should therefore be interpreted with caution, but suggest that the observed associations are unlikely to be fully explained by early-life vulnerability or pre-existing child characteristics.

We present here the estimates from further-adjusted Model 2, as well as those from the original Model 2, to allow for a better comparison.

Supplementary Table 15: Marginal structural model estimates of the effect of persistent high (≥60 dB(A)) versus low (<55 dB(A)) road traffic noise exposure across childhood on children's emotional and behavioral difficulties at 10·5 years - Further-adjusted model 2 (ELFE cohort, 2011–2022)

	**Model 2 (original)**		**Model 2 (further-adjusted on externalizing and internalizing difficulties at 5.5 years old)**	
**SDQ scale**	**β (95% CI)**	**P value**	**β (95% CI)**	**P value**
SDQ externalising symptoms	0.16 (-0.13, 0.45)	0.27	0.14 (-0.14, 0.43)	0.33
SDQ internalising symptoms	0.38 (0.14, 0.63)	<0.01	0.38 (0.13, 0.62)	<0.01
SDQ hyperactivity	0.13 (-0.07, 0.34)	0.20	0.12 (-0.08, 0.33)	0.24
SDQ conduct problems	0.03 (-0.10, 0.16)	0.68	0.02 (-0.11, 0.15)	0.74
SDQ emotional symptoms	0.20 (0.02, 0.37)	0.03	0.19 (0.02, 0.37)	0.03
SDQ peer relationship problems	0.19 (0.06, 0.32)	0.01	0.18 (0.05, 0.32)	0.01

Marginal estimates of exposure (≥60 dB(A)) at all three time points versus persistent low exposure (<55 dB(A)).

Estimates represent counterfactual contrasts between exposure histories in the weighted population and should not be interpreted as simple comparisons between observed exposure groups.

SDQ = Strengths and Difficulties Questionnaire, β = Adjusted regression coefficient, CI = Confidence Interval.

Supplementary Table 16: Marginal structural model estimates of the effect of childhood exposure to aircraft and railway noise (≥50 dB(A)) at any timepoint and children's emotional and behavioral difficulties at 10·5 years - Further-adjusted model 2 (ELFE cohort, 2011-2022)

	**Model 2 (original)**		**Model 2 (further-adjusted on externalizing and internalizing difficulties at 5.5 years old)**	
**SDQ scale**	**β (95% CI)**	**P value**	**β (95% CI)**	**P value**
**Aircraft noise**				
SDQ externalising symptoms	0.08 (-0.43, 0.59)	0.75	0.21 (-0.22, 0.63)	0.34
SDQ internalising symptoms	0.45 (-0.05, 0.94)	0.08	0.62 (0.17, 1.08)	0.01
SDQ hyperactivity	-0.08 (-0.42, 0.26)	0.65	-0.01 (-0.30, 0.29)	0.97
SDQ conduct problems	0.16 (-0.10, 0.42)	0.23	0.21 (-0.03, 0.46)	0.09
SDQ emotional symptoms	0.22 (-0.10, 0.54)	0.18	0.33 (0.02, 0.63)	0.04
SDQ peer relationship problems	0.23 (-0.06, 0.51)	0.12	0.30 (0.02, 0.57)	0.03
**Railway noise**				
SDQ externalising symptoms	-0.37 (-1.07, 0.32)	0.29	-0.33 (-0.83, 0.17)	0.19
SDQ internalising symptoms	-0.61 (-1.10, -0.12)	0.01	-0.47 (-0.90, -0.04)	0.03
SDQ hyperactivity	-0.22 (-0.73, 0.29)	0.40	-0.19 (-0.58, 0.20)	0.34
SDQ conduct problems	-0.15 (-0.42, 0.11)	0.26	-0.14 (-0.36, 0.08)	0.21
SDQ emotional symptoms	-0.41 (-0.73, -0.08)	0.01	-0.31 (-0.61, -0.01)	0.04
SDQ peer relationship problems	-0.21 (-0.47, 0.05)	0.12	-0.16 (-0.40, 0.09)	0.21

Estimates represent counterfactual contrasts between exposure histories in the weighted population and should not be interpreted as simple comparisons between observed exposure groups.

SDQ = Strengths and Difficulties Questionnaire, β = Adjusted regression coefficient, CI = Confidence Interval.

Section 13: Quantitative bias analysis for unmeasured confounding.

To indicate how strong an unmeasured confounder would need to be to explain the observed associations, we computed E-values based on odds ratios (results from Section 9), using a conversion to approximate risk ratios when outcome prevalence exceeded 15%. They were computed on the effect estimates and on the confidence interval limit closest to the null using the R package EValue. E-values for confidence intervals were not computed when the interval included the null value.

As shown in the table below, E-values were generally modest overall, but tended to be higher for statistically significant associations, indicating increased robustness and suggesting that moderately strong unmeasured confounding would be needed to explain these results.

Supplementary figure 6 illustrates the E-value for the association between road traffic noise and abnormal peer relationship problems SDQ subscale. The E-value of 1.24 indicates that an unmeasured confounder associated with both exposure and outcome by risk ratios of at least 1.24 each could explain away the observed association.

Supplementary Figure 6: E-value for the association between road traffic noise and abnormal peer relationship problems SDQ subscale

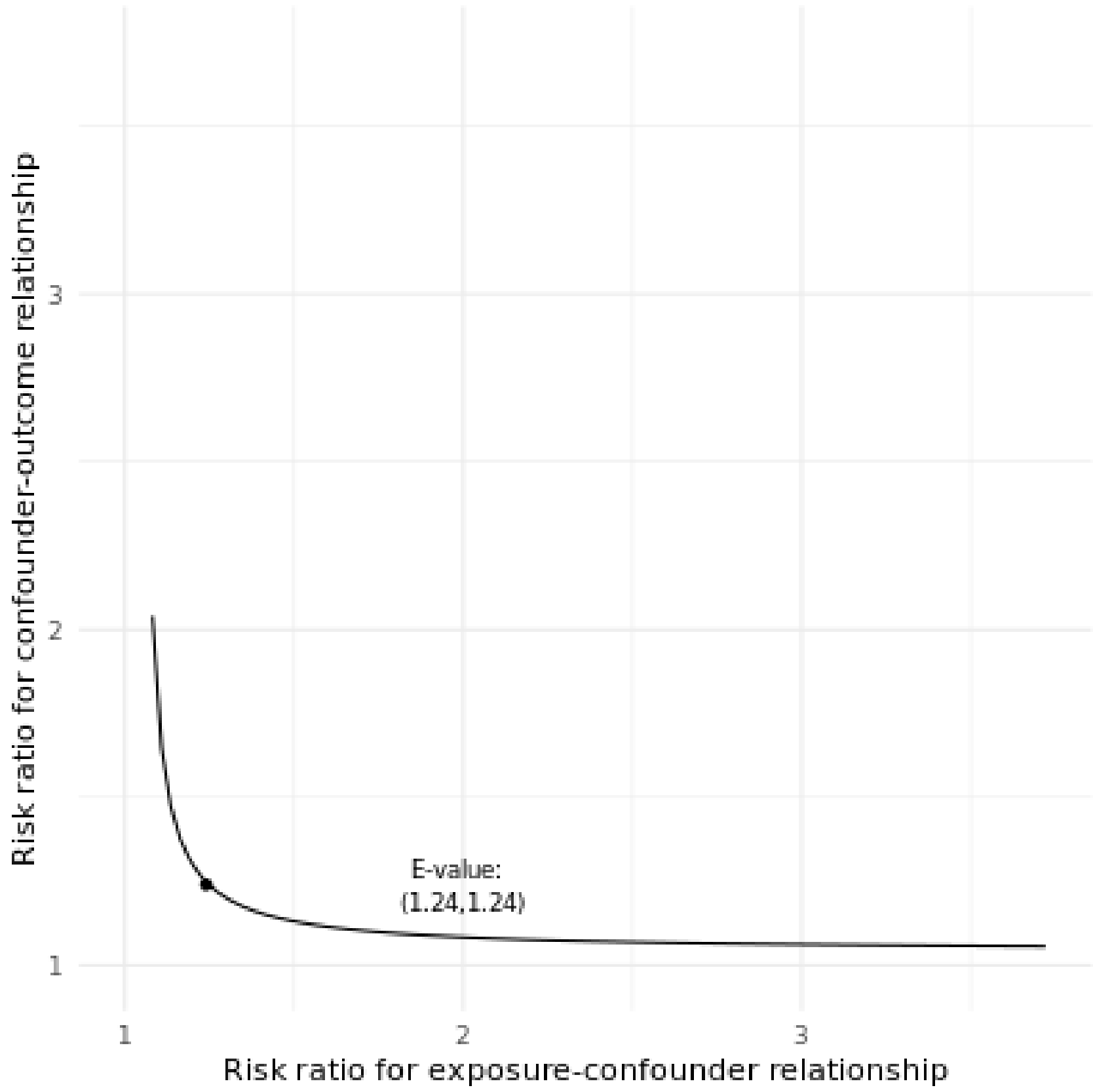

Supplementary Table 17: E-values based on MSM estimates of childhood exposure to road, aircraft and railway noise and children's emotional and behavioral difficulties at 10·5 years - considering thresholds of SDQ (ELFE cohort, 2011-2022)

**Predictor**	**Outcome**	**OR**	**P value**	**Prevalence (%)*** **	**E value point**	**E value CI**
Road traffic noise*	SDQ hyperactivity limit	1.02 (0.99, 1.06)	0.15	21.06	1,11	1
	SDQ hyperactivity abnormal	1.01 (0.98, 1.04)	0.41	14.00	1,11	1
	SDQ conduct problems limit	0.99 (0.95, 1.02)	0.43	24.74	1,08	
	SDQ conduct problems abnormal	1.01 (0.98, 1.04)	0.48	12.27	1,11	1
	SDQ emotional symptoms limit	1.03 (1.00, 1.07)	0.08	32.83	1,14	1
	SDQ emotional symptoms abnormal	1.02 (0.99, 1.06)	0.17	20.86	1,11	1
	SDQ peer relationship problems limit	1.04 (1.01, 1.08)	0.02	18.63	1,16	1,08
	SDQ peer relationship problems abnormal	1.04 (1.01, 1.07)	<0.01	9.77	1,24	1,11
Railway noise**	SDQ hyperactivity limit	0.93 (0.60, 1.44)	0.74	21.06	1,23	
	SDQ hyperactivity abnormal	0.89 (0.51, 1.57)	0.70	14.00	1,5	
	SDQ conduct problems limit	0.81 (0.51, 1.28)	0.37	24.74	1,46	
	SDQ conduct problems abnormal	0.85 (0.49, 1.46)	0.55	12.27	1,63	
	SDQ emotional symptoms limit	0.81 (0.55, 1.19)	0.29	32.83	1,46	
	SDQ emotional symptoms abnormal	0.60 (0.39, 0.92)	0.02	20.86	1,9	
	SDQ peer relationship problems limit	0.98 (0.59, 1.63)	0.94	18.63	1,11	
	SDQ peer relationship problems abnormal	0.69 (0.34, 1.41)	0.31	9.77	2,26	
Aircraft noise**	SDQ hyperactivity limit	0.72 (0.49, 1.06)	0.10	21.06	1,64	
	SDQ hyperactivity abnormal	0.73 (0.46, 1.15)	0.17	14.00	2,08	
	SDQ conduct problems limit	1.21 (0.87, 1.71)	0.26	24.74	1,43	1
	SDQ conduct problems abnormal	1.35 (0.88, 2.08)	0.17	12.27	2,04	1
	SDQ emotional symptoms limit	1.33 (0.96, 1.85)	0.09	32.83	1,57	1
	SDQ emotional symptoms abnormal	1.03 (0.70, 1.51)	0.89	20.86	1,14	1
	SDQ peer relationship problems limit	1.39 (0.96, 2.01)	0.08	18.63	1,64	1
	SDQ peer relationship problems abnormal	1.70 (1.09, 2.65)	0.02	9.77	2,79	1,4

Estimates represent counterfactual contrasts between exposure histories in the weighted population and should not be interpreted as simple comparisons between observed exposure groups.

OR = Odds Ratio.

*: MSM estimates of the effect of persistent high (≥60 dB(A)) versus low (<55 dB(A)) road traffic noise exposure across childhood on children's emotional and behavioral difficulties at 10·5 years - considering thresholds of SDQ.
**: MSM estimates of the effect of childhood exposure to aircraft and railway noise (≥50 dB(A)) at any timepoint and children's emotional and behavioral difficulties at 10·5 years - considering thresholds of SDQ.
***: Outcome prevalence. E-values were based on odds ratios, with a conversion to approximate risk ratios when outcome prevalence exceeded 15%.

E-values for confidence interval limits were not reported when the confidence interval included the null value, as no single bound could be identified as closest to the null.

To account for unmeasured housing characteristics (e.g., insulation, glazing, structural quality), we conducted a quantitative bias analysis using a proxy for housing disadvantage. We relied on national French data indicating that 2% of homeowners and 16% of renters reported insufficient or very insufficient housing conditions around the study period (2013) (1). Although this measure is self-reported and does not directly capture specific acoustic properties, it reflects overall housing quality and is plausibly associated with both environmental noise exposure and child mental health outcomes.

Based on these data, we defined plausible ranges for the prevalence of poor housing conditions from 2% to 10% among the unexposed and from 10% to 30% among the exposed, allowing for differential distribution across exposure groups. We also defined plausible ranges for the strength of its association with the outcome on the same scale as the marginal structural model estimates (0.1 to 0.5). For each combination of parameters, we computed the expected bias using a standard additive bias formula for linear models, defined as the product of the difference in confounder prevalence between exposure groups and the confounder–outcome association (bias = $\gamma \times [P(U=1|A=1) - P(U=1|A=0)]$). Bias-adjusted exposure effects were then obtained by subtracting this bias from the observed estimate. This approach assumes no interaction between the exposure and the unmeasured confounder and provides a transparent assessment of how varying assumptions about unmeasured confounding could influence the estimated association.

This analysis was performed on marginal structural model estimates of persistent high (≥60 dB(A)) versus low (<55 dB(A)) road traffic noise exposure across childhood (Table 3). The smallest significant coefficient is the effect on peer relationship problems (β=0.19, 95% CI 0.06 to 0.32, P=0.01 – Model 2). Smallest significant effect for railway/aircraft noise exposure is -0.41 (-0.73, -0.08) (Table 5), suggesting that similar robustness would be expected under the same sensitivity analysis.

Across all scenarios, bias-adjusted estimates remained consistently positive. Even under extreme assumptions combining high prevalence imbalance (30% vs 2%) and strong confounder–outcome associations ($\gamma = 0.5$), the association was only moderately attenuated and did not approach the null.

For the estimate of 0.19:

**Gamma**	**Beta min**	**Beta max**
0.1	0.162	0.19
0.3	0.106	0.19
0.5	0.05	0.19

References

1. Aumond P, Cariou S, Chiello O, Ecotière D, Bellec AL, Maltete D, et al. Couplage entre la base de données nationale PlaMADE et l'outil open-source NoiseModelling pour la réalisation de cartes de bruit stratégiques. 2022;

2. Bocher E, Bernard J, Wiederhold ELS, Leconte F, Petit G, Palominos S, et al. GeoClimate: a Geospatial processing toolbox for environmental and climate studies. Journal of Open Source Software. 29 sept 2021;6(65):3541.

3. Journal of Open Source Software [Internet]. [cité 5 févr 2026]. NoiseModelling: an open-source software for outdoor noise mapping. Disponible sur: https://joss.theoj.org/papers/95a66bc5f02dcc2a1fff7076d2991b63

4. Cole SR, Hernán MA. Constructing inverse probability weights for marginal structural models. Am J Epidemiol. 15 sept 2008;168(6):656-64.

5. Chesnaye NC, Stel VS, Tripepi G, Dekker FW, Fu EL, Zoccali C, et al. An introduction to inverse probability of treatment weighting in observational research. Clin Kidney J. 26 août 2021;15(1):14-20.

6. McCaffrey DF, Griffin BA, Almirall D, Slaughter ME, Ramchand R, Burgette LF. A Tutorial on Propensity Score Estimation for Multiple Treatments Using Generalized Boosted Models. Stat Med. 30 août 2013;32(19):3388-414.

7. Robins JM, Hernán MÁ, Brumback B. Marginal Structural Models and Causal Inference in Epidemiology. Epidemiology. sept 2000;11(5):550.

8. Goodman R, Meltzer H, Bailey V. The Strengths and Difficulties Questionnaire: a pilot study on the validity of the self-report version. Eur Child Adolesc Psychiatry. sept 1998;7(3):125-30.
